# Diet‐Related Metabolites Associated with Cognitive Decline Revealed by Untargeted Metabolomics in a Prospective Cohort

**DOI:** 10.1002/mnfr.201900177

**Published:** 2019-07-09

**Authors:** Dorrain Yanwen Low, Sophie Lefèvre‐Arbogast, Raúl González‐Domínguez, Mireia Urpi‐Sarda, Pierre Micheau, Melanie Petera, Delphine Centeno, Stephanie Durand, Estelle Pujos‐Guillot, Aniko Korosi, Paul J Lucassen, Ludwig Aigner, Cécile Proust‐Lima, Boris P Hejblum, Catherine Helmer, Cristina Andres‐Lacueva, Sandrine Thuret, Cécilia Samieri, Claudine Manach

**Affiliations:** ^1^ Human Nutrition Unit INRA, Université Clermont Auvergne F‐63000 Clermont‐Ferrand France; ^2^ Bordeaux Population Health Research Center Inserm, University of Bordeaux UMR 1219 F‐33000 Bordeaux France; ^3^ Nutrition, Food Science and Gastronomy Department, Faculty of Pharmacy and Food Science, CIBER Fragilidad y Envejecimiento Saludable (CIBERFES) Instituto de Salud Carlos III University of Barcelona Av Joan XXIII 27–31 08028 Barcelona Spain; ^4^ Université Clermont Auvergne INRA, UNH, Plateforme d'Exploration du Métabolisme MetaboHUB Clermont F‐63000 Clermont‐Ferrand France; ^5^ Brain Plasticity Group, SILS‐CNS University of Amsterdam Science Park 904 1098 XH Amsterdam The Netherlands; ^6^ Institute of Molecular Regenerative Medicine, Spinal Cord Injury and Tissue Regeneration Center Salzburg, Paracelsus Medical University Salzburg 5020 Austria; ^7^ Inria, SISTM, Bordeaux Sud‐Ouest F‐33000 Bordeaux France; ^8^ Department of Basic and Clinical Neuroscience Maurice Wohl Neuroscience Institute Institute of Psychiatry, Psychology and Neuroscience, King's College London London SE5 9NU UK

**Keywords:** aging, coffee, cognitive decline, dietary biomarkers, untargeted metabolomics

## Abstract

**Scope:**

Untargeted metabolomics may reveal preventive targets in cognitive aging, including within the food metabolome.

**Methods and results:**

A case‐control study nested in the prospective Three‐City study includes participants aged ≥65 years and initially free of dementia. A total of 209 cases of cognitive decline and 209 controls (matched for age, gender, education) with slower cognitive decline over up to 12 years are contrasted. Using untargeted metabolomics and bootstrap‐enhanced penalized regression, a baseline serum signature of 22 metabolites associated with subsequent cognitive decline is identified. The signature includes three coffee metabolites, a biomarker of citrus intake, a cocoa metabolite, two metabolites putatively derived from fish and wine, three medium‐chain acylcarnitines, glycodeoxycholic acid, lysoPC(18:3), trimethyllysine, glucose, cortisol, creatinine, and arginine. Adding the 22 metabolites to a reference predictive model for cognitive decline (conditioned on age, gender, education and including ApoE‐ε4, diabetes, BMI, and number of medications) substantially increases the predictive performance: cross‐validated Area Under the Receiver Operating Curve = 75% [95% CI 70–80%] compared to 62% [95% CI 56–67%].

**Conclusions:**

The untargeted metabolomics study supports a protective role of specific foods (e.g., coffee, cocoa, fish) and various alterations in the endogenous metabolism responsive to diet in cognitive aging.

## Graphical abstract

**The Three‐City cohort of older persons with a 12‐year follow‐up for cognition** is leveraged to identify, in the serum of initially non‐demented participants, metabolites associated with subsequent cognitive decline. Untargeted metabolomics reveals a signature of 22 metabolites improving cognitive decline prediction. It includes diet‐derived metabolites (e.g., from coffee, citrus juice, cocoa, fish), as well as endogenous metabolites responsive to diet.

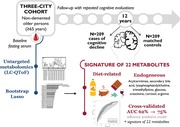

## 1 Introduction

Cognitive aging is a major public health concern worldwide. Dementia and its main form Alzheimer's disease (AD) are leading causes of accelerated cognitive decline (CD), however are lacking etiological treatment. As pathological processes are thought to evolve over years before CD becomes apparent, early prevention through diet management may be of critical importance.^1^, ^2^ Various plant‐based healthy dietary patterns have been related to a lower risk of dementia and CD;^3^ yet, only a few nutritional bioactives (e.g., long‐chain omega‐3 fatty acids, vitamins, carotenoids, and polyphenols)^4^ have been linked to cognitive aging, and there is currently no consensus on a gold‐standard nutrition‐based preventive strategy against cognitive aging and dementia. With habitual diet providing up to 25 000 compounds and additional thousands of host‐ and gut microbiota‐derived metabolites,^5^ most interesting nutritional compounds that may promote brain health likely remain undiscovered.

Metabolomics may enable the identification of new pathways and preventive targets in cognitive aging and dementia, particularly within the food metabolome, that is, the part of the human metabolome derived directly from food digestion. Metabolomics provides a global picture of individuals’ biological status, as it simultaneously measures a wide profile of metabolites in biofluids, including diet‐derived metabolites and endogenous metabolites modulated by dietary intake.^6^ Metabolomics has the potential to capture the complexity of dietary exposures and their impact on metabolism, taking into account inter‐individual variability.^5^

Epidemiological studies investigating metabolic changes in dementia have only recently emerged, identifying new biomarkers of diagnosis and prognosis.^7^, ^8^, ^9^, ^10^ However, most of these studies used a targeted metabolomics approach (i.e., assessing a list of known metabolites, generally from the endogenous metabolome),^9^, ^10^ thereby missing potential new metabolites from the food metabolome. Moreover, these studies were cross‐sectional or used a short‐term prospective design where variations in the metabolome may reflect underlying diseases. Lastly, very few studies have investigated the trajectory of cognitive aging from a long‐term perspective, which may be a more powerful approach to capture predictors of both early (preclinical) dementia stages and normal cognitive aging.

We therefore applied untargeted metabolomics analysis on the serum of participants from a large, well‐established cohort on dementia, who were initially free of dementia at the time of blood draw (baseline) and provided repeated measures of cognition over 12 years, to identify an early serum metabolomics signature of subsequent CD.

## 2 Experimental Section

### 2.1 Population

The Three‐City (3C) study is a French population‐based cohort on dementia initiated in 1999–2000, including 9294 non‐institutionalized older persons aged ≥65 years selected from the electoral rolls of three cities (Bordeaux [*n* = 2104], Dijon [*n* = 4931], and Montpellier [*n* = 2259]).^11^ The Consultative Committee for the Protection of Persons participating in Biomedical Research at Kremlin‐Bicêtre University Hospital (Paris, France) approved the 3C study protocol and all participants provided written consent. At enrollment, face‐to‐face interviews were conducted to collect socio‐demographic and lifestyle characteristics (including a food frequency questionnaire (FFQ)), medical information, cognitive testing, blood pressure and anthropometric measurements, and fasting blood samples for constitution of a biobank. Follow‐up visits were performed every two to three years, including in‐person neuropsychological assessments carried out by a trained psychologist. Clinical diagnosis of dementia was established and validated by an independent committee of neurologists, using the Diagnostic and Statistical Manual of Mental Disorders, fourth edition.^12^

### 2.2 Study Design

A case‐control study nested within the 3C‐Bordeaux cohort was constructed to investigate the relationships between variations in serum metabolome and subsequent CD. 1293 participants not diagnosed with dementia at baseline, with available serum samples in the biobank and with at least one repeated cognitive evaluation over 12 years, were retained for case‐control sampling (Figure S1, Supporting Information). Then, individual slopes of cognitive change estimated by a linear mixed model were used. The primary outcome was the change in a composite score of global cognition defined as the average of Z‐scores of five neuropsychological tests at each follow‐up (Mini‐Mental State Examination, Benton Visual Retention Test, Isaac's Set Test, Trail‐Making Test part A, Trail‐Making Test part B; see Method S1, Supporting Information, for details). Cases were defined as participants with the worst slopes of CD and controls as those with CD below median value (i.e., >median slope). Finally, 209 cases with greater CD were successfully matched (based on age at baseline, gender and educational level) to 209 controls with slower CD, leading to a total sample size of *n* = 418 subjects.

### 2.3 Covariates

At baseline, regularly consumed medications were recorded and cardio‐metabolic risk factors were assessed, including BMI (kg m^−^²), diabetes (fasting glucose ≥7.2 mmol L^−1^ or specific medication) and fasting plasma levels of glucose, cholesterol, and triglycerides (measured by routine enzymatic methods). ApoE‐ε4 genotype was defined as carrying at least one ε4 allele vs absence of ε4 allele. Lifestyle factors included regular physical activity ([yes/no] defined as having either an intensive leisure activity [e.g., swimming] ≥1 h/week or a moderate activity [e.g., walking or household] ≥1 h per day); smoking status; alcohol consumption; regular consumption of main food/beverage groups. For physical activity, data were missing in 17% of the samples and a specific missing category was created. For all other covariates, missing values were <2% of the samples and the reference category was assigned to missing data (for categorical variables) or the median value (for continuous variables).

### 2.4 Dietary Data

Dietary habits were primarily assessed using a brief FFQ administered concomitantly to blood sampling at baseline, which recorded the frequency of intakes of 10 broad food/beverages^13^ and the number of glasses/cups per day of alcohol, wine, coffee, and tea. For foods of specific interest to the study and not ascertained in this FFQ (e.g., juices) and for specific nutrients/food compounds (e.g., polyphenols), a more comprehensive FFQ and a 24‐h dietary recall administered in a subsample from 3C Bordeaux in 2001–2002 (data available for 351 participants out of the 418 subjects of the case‐control study; see Method S1, Supporting Information, for details) was used.

### 2.5 Sample Preparation and Untargeted LCMS Metabolomics

A detailed description of LCMS data acquisition and processing is outlined in Method S2, Supporting Information. Briefly, fasting baseline serum samples were de‐proteinized with acidified methanol. Metabolic profiles were acquired on a 22‐min elution gradient on an U300 UHPLC system (Thermo Scientific) coupled to a high resolution QTOF (Bruker Impact HD2) operating in positive electrospray ionization mode, with a scan range from 50 to 1000 *m*/*z*. Quality controls were injected every ten samples to monitor stability of the analytical system and allow signal drift and batch effect correction.

LCMS data was processed using Galaxy WorkFlow4Metabolomics, an open access web‐based platform (http://workflow4metabolomics.org/). Parameters used for peak detection, grouping, retention time correction, quality checks, and signal drift correction are detailed in Table S1, Supporting Information. A matrix of 1136 ions characterized by a retention time, *m*/*z*, and relative intensity was obtained and used for correlation analyses. Ions annotated as adducts, isotopes, or fragments of the same metabolite or with high analytical variability or too low intensity according to predefined thresholds were discarded, leaving a matrix of 301 ions for LASSO regression analysis.

### 2.6 Statistical Analyses

In primary analyses, least absolute shrinkage and selection operator (LASSO) conditional logistic regression was used to select a set of metabolites associated with the odds of developing CD over 12 years in the case‐control study (Method S3, Supporting Information).^14^ The model was conditioned on matching variables (age, gender, and education), and adjusted for BMI and total number of medications regularly consumed. As LASSO regression may lead to unstable solutions, bootstrap resampling was used to enhance the robustness of variable selection.^15^ LASSO‐penalized conditional logistic regressions were repeated on 1000 bootstrapped samples; the metabolites were ordered by decreasing percentage of selection across bootstraps and focused on those selected in >40% of bootstraps to define a serum metabolomics signature of CD.

Several additional analyses were conducted. First, an un‐penalized conditional logistic regression was ran to estimate the unbiased multivariable adjusted odds ratio (OR) of each selected metabolite for greater versus slower CD (confidence intervals were not estimated as known to be biased in post‐selection inference).^16^ Second, the predictive ability of the signature compared to a reference predictive model conditioned on age, gender, and education (the matching variables) and including: (i) covariates from the selection model (BMI and medication use), and (ii) ApoE‐ε4 genotype and diabetes, two additional important predictors of CD, was assessed. The added value of the signature was evaluated for prediction of CD by comparing Area Under the Receiver Operating Characteristic Curves^17^ between the reference predictive model and a model additionally including the metabolomics signature. Area Under the Curves (AUC) were computed using a leave‐pair‐out‐cross‐validation and confidence intervals were obtained from 1000 bootstraps.

Moreover, several supplementary analyses were conducted to assist in metabolite identification and interpretation of findings. Pearson correlations of the 22 metabolites were estimated from the signature with food/nutrient intakes assessed by the brief baseline FFQ or the comprehensive FFQ and 24‐h dietary recall collected two years later (Method S1, Supporting Information). These analyses were exploratory as they were based on subsamples with relevant dietary data available. Pearson correlations between intakes of coffee and hydroxycinnamates and the intensities of all ions (*n* = 1136) of metabolomic profiles were also estimated. Analyses were controlled for the False Discovery Rate (FDR) using Benjamini–Hochberg procedure. Pearson correlations between ions of interest were also examined. Statistical analyses were performed using the R software version 3.3.2. Penalized and un‐penalized conditional logistic regressions were fitted as stratified discrete‐time Cox proportional hazards models using the penalized and survival R packages, respectively.

### 2.7 Metabolite Identification

In untargeted metabolomics, the chemical identity of the detected ions is a priori unknown. The multi‐step identification process is described in Method S2, Supporting Information. Briefly, in‐house and online databases were queried to obtain hypotheses of identification based on accurate mass. Custom‐curated databases of known biomarkers of intake for specific foods, endogenous compounds associated with cognition and the Bordeaux 3C medication list were also used for drawing hypotheses of high biological plausibility. MS/MS fragmentation analyses were performed on the Bruker QTOF and on an ultra‐high resolution spectrometer LTQ Orbitrap (Velos, Thermo‐Scientific). Fragmentation spectra were compared to those available in databases or in the literature, or to in silico predicted fragmentation pathways generated with MassFrontier (Thermo‐Scientific). Formal identification (level 1, as described by Sumner et al.)^18^ was obtained by matching of masses, fragmentation pattern, and retention time to an authentic standard. When standards were not available, putative identification was obtained by comparison to analytical data reported in online databases or in the literature (for a compound [level 2] or a class of compounds [level 3]).

## 3 Results

The participants were 76 years old on average at baseline; 66% were female and 29% had reached secondary school or over (**Table** 1). Participants were followed for cognition for an average 8.5 years (SD = 2.6). All participants were initially free of dementia at the time of blood sampling; among cases of CD, 51% developed dementia during follow‐up, versus 3% in the control group (results not shown in the tables). Average BMI, plasma cholesterol, and triglycerides were similar between groups at baseline (*p* ≥ 0.12). In contrast, compared to controls, cases with greater CD consumed a higher number of medications, had higher blood glucose and were more often diabetics, carried more often the ApoE‐ε4 allele and practiced less regularly exercise (all *p* < 0.02). The frequency of consumption of main food groups were similar between groups except for chocolate that was more frequently consumed among controls (*p* < 0.01). Differences in intakes of more detailed food groups and nutrient/food compounds is provided in Table S2, Supporting Information.

**Table 1:** Baseline characteristics of cases of cognitive decline (*n* = 209) and controls with slower cognitive decline (>median slope, *n* = 209) in a case‐control study matched for age, gender and education, nested within the 3C Bordeaux cohort

	Cases	Controls	*p* a
**Sociodemographic characteristics**			
Age (years)	75.9 (4.5)	75.7 (4.2)	–
Gender, female	66.0	66.0	–
Education, ≥secondary school	28.7	28.7	–
**Health indicators**			
Number of drugs consumed	4.9 (2.7)	4.1 (2.4)	<0.01
BMI (kg m^−^²)	26.8 (4.4)	26.1 (3.6)	0.12
Plasma total cholesterol (mmol L^−1^)	5.8 (0.9)	5.8 (1.0)	0.98
Plasma triglycerides (mmol L^−1^)	1.4 (0.8)	1.3 (0.6)	0.23
Plasma glucose (mmol L^−1^)	5.4 (1.6)	5.1 (1.0)	0.015
Diabetes	13.2	5.7	0.02
ApoE‐ε4 carrier	26.2	12.0	<0.01
**Lifestyle characteristics**			
Regular Physical activity	27.8	38.3	<0.01
Smoking status			0.75
Never	67.5	65.1	
Former	27.8	30.6	
Current	9.1	4.3	
*Beverage intakes*			
Alcoholic beverages (glasses/week)	9.4 (10.5)	10.8 (13.2)	0.20
Wine (glasses/week)	8.4 (9.5)	9.3 (11.5)	0.35
Regular coffee consumption (daily)	75.1	78.9	0.37
Regular tea consumption (daily)	25.4	22.0	0.39
*Food groups, regular intakes*			
Dairy products (daily)	94.7	93.3	0.53
Meat (≥4 times/week)	65.6	62.2	0.47
Fish (≥2 times/week)	54.1	56.0	0.68
Eggs (≥2 times/week)	40.7	43.5	0.55
Cereal (daily)	93.8	94.7	0.68
Raw fruit (daily)	82.8	83.7	0.80
Raw vegetables (daily)	44.0	52.2	0.11
Cooked fruit and vegetables (daily)	72.7	77.5	0.29
Legumes (≥1 time/week)	30.1	28.7	0.75
Chocolate (≥2 times/week)	38.8	52.4	<0.01

Values are mean (SD) or percentages of non‐missing values. ApoE‐ε4, allele ε4 for the apolipoprotein E gene; HDL, high‐density lipoprotein; LDL, low‐density lipoprotein; BMI, body mass index

a Estimated using conditional logistic regression.

### 3.1 Identification of a Set of Serum Metabolites Associated with Subsequent Cognitive Decline

**Figure 1:**
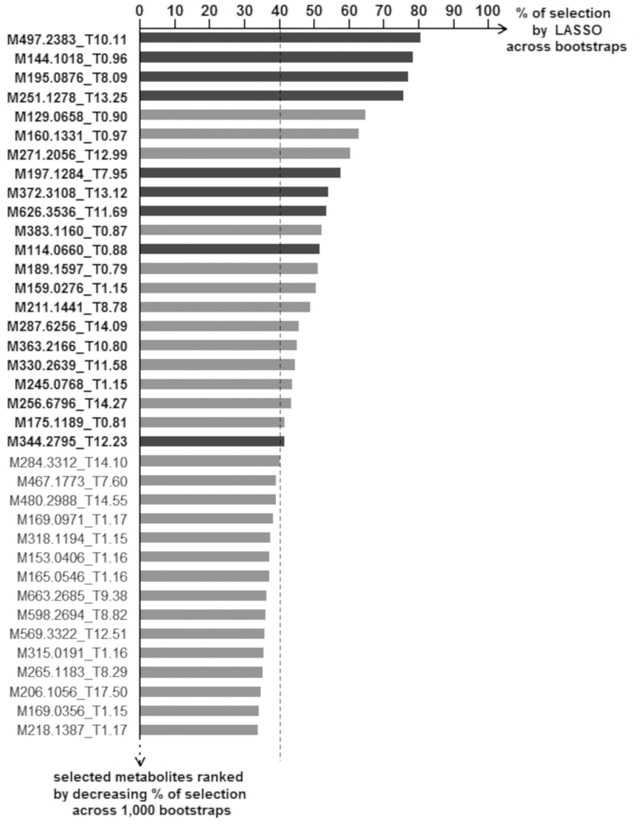
Identification of a metabolomics signature of cognitive decline in the 3C Bordeaux cohort (*n* = 418). We applied LASSO‐penalized conditional logistic regression on 1000 bootstrap samples to identify the ions/metabolites robustly associated with the odds of cognitive decline in the case‐control study. Ions/metabolites are ranked by decreasing frequency of selection across bootstraps. Dark grey bars indicate metabolites selected on the initial sample, and light grey bars those selected on bootstrapped samples only. We retained the 22 ions/metabolites selected in >40% of bootstraps (with names highlighted in bold font). For each bootstrapped model, the optimal penalization was chosen by leave‐pair‐out cross‐validation. Models were conditioned on the matching variables (age, gender, and level of education) and adjusted for body mass index and the number of medications regularly consumed. Ion/metabolites are defined with their mass‐to‐charge ratio (M) and retention time (T).

The bootstrap‐enhanced LASSO procedure identified a set of 22 ions robustly associated with CD (**Figure** 1). The multivariate ORs and identification of these ions are presented in **Table** 2. A complete description of the analytical data (*m*/*z* values, retention times, elementary composition and MS/MS fragments) and associated information supporting identification of the 22 metabolites are given in Table S3, Supporting Information and Figure S2, Supporting Information. The serum metabolomics signature included food‐derived metabolites (atractyligenin glucuronide, proline betaine, caffeine, 3‐carboxy‐4‐methyl‐5‐pentyl‐2‐furanpropionic acid (CMPFP), cyclo(prolyl‐valyl), cyclo(leucyl‐prolyl)), ten endogenous metabolites (myristoylcarnitine, glycodeoxycholic acid 3‐glucuronide (GDCA), glucose, creatinine, N‐trimethyllysine, 1‐linolenoyl‐*sn*‐glycero‐3‐phosphocholine (lysoPC(18:3)), cortisol, undecanoylcarnitine/4,8 dimethylnonanoylcarnitine, arginine, lauroylcarnitine) and six unidentified metabolites (Table 2).

**Table 2:** Multivariate associations between the 22 serum ions/metabolites selected in the metabolomics signature and the odds of subsequent cognitive decline, in the 3C Bordeaux cohort (*n* = 418)

Selection rank	Ion	Metabolite name	Identification level	OR
1	M497.2383_T10.11	Atractyligenin glucuronide	2	0.72
2	M144.1018_T0.96	Proline betaine	1	1.56
3	M195.0876_T8.09	Caffeine	1	1.75
4	M251.1278_T13.25	CMPFP	2	0.74
5	M129.0658_T0.90	–	4	1.49
6	M160.1331_T0.97	–	4	1.83
7	M271.2056_T12.99	–	4	0.69
8	M197.1284_T7.95	Cyclo(prolyl‐valyl)	1	0.90
9	M372.3108_T13.12	Myristoylcarnitine	1	1.01
10	M626.3536_T11.69	GDCA	1	1.12
11	M383.1161_T0.87	Glucose	1	1.24
12	M114.0660_T0.88	Creatinine	1	1.41
13	M189.1597_T0.79	N‐trimethyl‐Lysine	1	1.09
14	M159.0276_T1.15	–	4	1.40
15	M211.1441_T8.78	Cyclo(leucyl‐prolyl)	1	0.68
16	M287.6256_T14.09	LysoPC(18:3)	2	0.76
17	M363.2166_T10.80	Cortisol	1	0.74
18	M330.2639_T11.58	Undecanoylcarnitine/4,8 dimethylnonanoylcarnitine	3	0.83
19	M245.0768_T1.15	–	4	1.29
20	M256.6796_T14.27	–	4	0.85
21	M175.1189_T0.81	L‐Arginine	1	1.29
22	M344.2795_T12.23	Lauroylcarnitine	1	1.32

Ions/metabolites are ordered by decreasing frequency of selection across bootstraps and referred to by mass‐to‐charge‐ratio (M) and retention time (T). Odds Ratios (ORs) for cognitive decline were estimated using a conditional logistic regression conditioned on matching variables (age, gender and educational level) and adjusted for BMI and number of medications regularly consumed. ORs are for 1SD‐increment of metabolite intensity. Confidence intervals are not valid in post‐selection inference and hence were not estimated. Level of identification is assigned as: 1, identification validated with standards; 2, putative identification by comparison with databases or literature; 3, putative identification of a chemical class; 4, unknown. CMPFP, 3‐carboxy‐4‐methyl‐5‐pentyl‐2‐furanpropionic acid; GDCA, glycodeoxycholic acid‐3‐glucuronide; LysoPC(18:3), 1‐linolenoyl‐sn‐glycero‐3‐phosphocholine.

**Figure 2:**
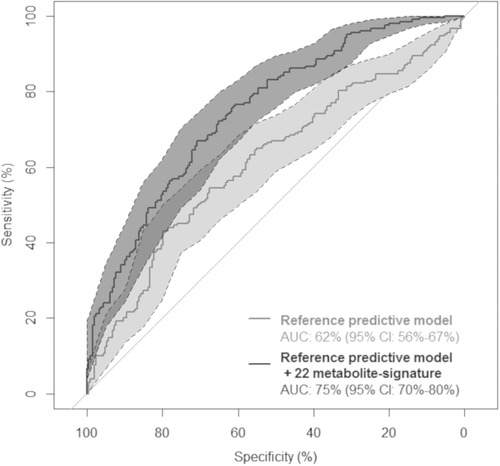
Cross‐validated ROC curves for a reference predictive model for cognitive decline (light grey curve) and a model additionally including the 22 metabolite‐signature (dark grey curve), the 3C Bordeaux cohort (*n* = 418). Areas Under the Curve (AUC) were estimated using conditional logistic regressions conditioned on age at baseline, gender and level of education and adjusted for body mass index, number of medications, ApoE‐ε4 genotype and diabetes. ROC curves and AUCs were estimated by leave‐pair‐out cross‐validation; confidence intervals for AUC were computed from 1000 bootstraps.

Adding the 22 metabolite‐signature to a reference predictive model (conditioned on age, gender and education, and including ApoE‐ε4, diabetes, BMI and number of medications) increased the predictive performance for cognitive decline from a cross‐validated AUC of 62% (95% CI 56–67%) to 75% (95% CI 70–80%) (**Figure** 2).

#### 3.1.1 Food‐Derived Metabolites

The metabolites with the highest rate of selection (>75%, Figure 1) were food‐derived metabolites. The first‐ranked compound (Table 2) was atractyligenin glucuronide (*m*/*z* 497.2383), a diterpenoid metabolite previously reported as a robust biomarker of coffee intake.^19^ Atractyligenin glucuronide was associated with a lower odds of CD (multivariable‐adjusted OR = 0.72). Consistently, cyclo(leucyl‐prolyl) (*m*/*z* 211.1441, 15th‐ranked in bootstrap selection), another known biomarker of coffee intake,^19^, ^20^ was inversely associated with CD (OR = 0.68). In contrast, caffeine (*m*/*z* 195.0876, third‐ranked) was unexpectedly associated with higher odds of CD (OR = 1.75).

The second‐ranked metabolite was identified as proline betaine (*m*/*z* 144.1018), a biomarker of citrus intake^21^ that was found associated with increased odds of CD (OR = 1.56). Three other food‐derived metabolites of the signature were inversely associated with CD: i) a metabolite tentatively identified as CMPFP (*m*/*z* 251.1278, fourth‐ranked: OR = 0.74), a structural derivative of a reported fish biomarker, 3‐carboxy‐4‐methyl‐5‐propyl‐2‐furanopropionic acid (CMPF),^22^ ii) an unknown metabolite (*m*/*z* 271.2056, seventh‐ranked; OR = 0.69) possibly linked to red wine intake (see below correlations with dietary intake), and iii) cyclo(prolyl‐valyl) (*m*/*z* 197.1284; eight‐ranked; OR = 0.90), a diketopiperazine reported in chocolate, and other fermented and heated foods.^23^

#### 3.1.2 Endogenous Metabolites

Three endogenous metabolites were associated with lower odds of CD, including lysoPC(18:3) (*m*/*z* 287.6256; 16th‐ranked; OR = 0.76), cortisol (*m*/*z* 363.2166; 17th‐ranked; OR = 0.74) and a medium‐chain acylcarnitine (undecanoylcarnitine or 4,8 dimethylnonanoylcarnitine, *m*/*z* 330.2639, 18th‐ranked; OR = 0.83). In contrast, six of the endogenous metabolites were associated with greater odds of CD, including GDCA (*m*/*z* 626.3536; 10th‐ranked; OR = 1.12), glucose (*m*/*z* 383.1160; 11th‐ranked; OR = 1.24), creatinine (*m*/*z* 114.0660; 12th‐ranked; OR = 1.41), trimethyllysine (*m*/*z* 189.1597; 13th‐ranked; OR = 1.09), L‐arginine (*m*/*z* 175.1189; 21st‐ranked; OR = 1.29), and lauroylcarnitine (*m*/*z* 344.2795; 22nd‐ranked; OR = 1.32). Myristoylcarnitine (*m*/*z* 372.3108; ninth‐ranked; OR = 1.01) were less clearly associated with CD.

Associations of the 22 metabolites to CD were generally unchanged (or slightly strengthened) when further adjusting for ApoE‐ε4, diabetes and physical activity. Only the association of glucose to greater CD was attenuated when adjusting for diabetes, as expected; and the association of myristoylcarnitine with lower CD arose when further adjusting for ApoE‐ε4 status and physical activity (OR = 0.81).

Despite our efforts, six ions (*m*/*z* 129.0658, 160.1331, 271.2056, 159.0276, 245.0768, and 256.6796) remain unidentified. Their spectral data are provided in Figure S2, Supporting Information.

### 3.2 Associations between the 22 Serum Metabolites of the Signature and Food/Nutrient Intakes

In supplementary analyses, we examined the correlations between the 22 serum metabolites from the signature and food/nutrient intakes recorded in the 3C cohort (see Figure S3, Supporting Information, for heatmap of correlations). Two metabolites, atractyligenin glucuronide, and cyclo(leucyl‐prolyl), were correlated with coffee intake (*r* = 0.39 and *r* = 0.37 respectively) and hydroxycinnamates (*r* = 0.25, 0.36), while unexpectedly, caffeine was not (*r* = 0.15 and 0.14, *p* > 0.08). Moreover, caffeine was not significantly correlated with other dietary sources of caffeine, including tea and chocolate (*p* > 0.13), while soda drinks, a known source of caffeine, were not examined as poorly consumed in this older population. To provide additional insight on the relationship between coffee and CD in our cohort, we analyzed the correlations between all ions of the untargeted metabolomics profile (irrespective of the signature) and the intakes of coffee and its major polyphenol class, hydroxycinnamates. Five ions/metabolites were significantly correlated with coffee intake, including atractyligenin glucuronide and cyclo(leucyl‐prolyl) that had the strongest correlations (Figure S4, Supporting Information). The ion (*m*/*z* 96.0444, 1.17 min, unknown) showed high correlations with coffee intake (*r* = 0.39), hydroxycinnamates (*r* = 0.26), atractyligenin glucuronide (*r* = 0.55), and cyclo(leucyl‐prolyl) (*r* = 0.38). This unidentified ion (Figure S2, Supporting Information ) was not in the signature but had a univariate odds ratio for CD of 0.90 (for 1SD‐increase in intensity). Paraxanthine (*m*/*z* 181.0720, RT 7.44 min, level 1), the major metabolite of caffeine, and trigonelline (*m*/*z* 138.0535, RT 0.93 min, level 1), previously proposed as a biomarker of coffee intake,^24^ but also of legumes,^25^ were modestly correlated with coffee intake (*r* = 0.26 and *r* = 0.18) and showed no association with CD (univariate OR for 1SD‐increase = 0.98 and 1.03, respectively). In conclusion, atractyligenin glucuronide, cyclo(leucyl‐prolyl), and *m*/*z* 96.0444 were the best markers of coffee intake and were all associated with a reduced risk of CD while caffeine, paraxanthine, and trigonelline reflected coffee intake much less accurately and were inconsistently associated with CD.

Proline betaine was correlated with citrus juice intake (*r* = 0.32, *p* < 0.001), total citrus (*r* = 0.27, *p* < 0.001), citrus‐specific flavanones (*r* = 0.28, *p* < 0.001), and vitamin C intake (*r* = 0.20, *p* = 0.011) but not with citrus fruit intake (*r* = 0.13, *p* = 0.34). CMPFP was not correlated with any food/nutrient (Figure S3, Supporting Information). We found a positive correlation between CMPFP and CMPF in our metabolomics profiles (*r* = 0.37). Both compounds are gut microbial metabolites of furan fatty acids that are found in high amounts in marine fish.^26^ CMPF, however not CMPFP, was correlated with fish intake (*r* = 0.27, *p* < 0.001, and *r* = −0.02, *p* = 0.93, respectively).

One unidentified compound (*m*/*z* 271.2056) correlated with wine intake (*r* = 0.34), red wine intake (*r* = 0.25), caloric intake (*r* = 0.19), polysaccharides (*r* = 0.21), alcohol (*r* = 0.27), and major wine polyphenols including dihydroflavonols (*r* = 0.25), proanthocyanidins (PACs, *r* = 0.19), stilbenes (*r* = 0.26), and lignans (*r* = 0.24) (all *p* ≤ 0.02). Cyclo(prolyl‐valyl) correlated with chocolate intake (*r* = 0.22, *p* < 0.001), PACs (*r* = 0.20, *p* = 0.001), the major polyphenols of cocoa, and serum levels of theobromine (*r* = 0.65, *p* < 0.001), a purine alkaloid primarily found in chocolate in Western diets.^27^ These data suggest that cyclo(prolyl‐valyl), known as an abundant diketopiperazine in roasted cocoa beans,^28^ may represent a new candidate biomarker of cocoa intake. This also suggests that chocolate consumption could be associated with slower CD in our study. Interestingly, theobromine was correlated with chocolate intake (*r* = 0.20) and although not selected in the 22 metabolite‐signature, was found negatively associated to CD (univariate OR for 1SD‐increase = 0.85).

## 4 Discussion

Using untargeted metabolomics, we identified in the serum of older persons free of dementia, 22 metabolites associated with subsequent CD over 12 years. Several metabolites were related to coffee intake including atractyligenin glucuronide and cyclo(leucyl‐prolyl), two biomarkers of coffee intake, that clearly suggest a protective association of coffee consumption with CD in this older population. There is biological and epidemiological evidence supporting a protective role of coffee on neurological function. Observational studies, including the 3C study,^29^ have reported associations between coffee intake and a lower risk of age‐related cognitive disorders, although prospective cohort studies are still limited in number and have showed inconsistent results.^30^, ^31^ Among the vast number of coffee compounds, polyphenols (e.g., chlorogenic acids), diterpenes, trigonelline, melanoidins, and caffeine may exert cell signaling, prebiotic, antioxidant, anti‐inflammatory, antihypertensive, hypoglycemic, vasculoprotective, and neurostimulant activities.^32^, ^33^ Many of these compounds could contribute to the neuroprotective effects of coffee including those bioactives with a short pharmacokinetic profile during the postprandial phase and hence not detected in the fasting serum samples in our study. Unexpectedly, we found an inconsistent association of serum caffeine with greater odds of CD. A protective effect of caffeine on CD has been suggested,^29^, ^30^, ^31^, ^32^ although the body of evidence is weak and coffee effect may have been unduly attributed to caffeine. Many investigators used the number of cups of coffee consumed to assess caffeine exposure. Yet, circulating levels of caffeine do not depend only on the quantity ingested but are largely affected by genetic polymorphisms (e.g., CYP1A2) and co‐ingestion of drugs (e.g., cardiovascular medications, estrogen replacement therapy).^34^ The possible negative effect of caffeine in some population subgroups deserves further investigation. It may be linked to interactions with drugs or bioactive compounds, or to genetic polymorphisms affecting caffeine bioactivity on specific targets such as adenosine receptor A2.^34^ Finally, our findings support a protective effect of coffee, while caffeine was not the major bioactive compound involved and may even have counteracted the beneficial activity of other coffee compounds in some individuals. Future research with accurate measurement of all coffee‐derived metabolites in blood should provide a better understanding of components actually involved in coffee protective effects.

Proline betaine, a well‐validated biomarker of citrus intake,^21^, ^35^ was another important food‐derived metabolite found associated with greater CD in this study. This observation is in agreement with a smaller metabolomics study that identified proline betaine among the increased serum metabolites in AD patients,^36^ Yet, it contrasts with the reported beneficial role of citrus fruit, which are good sources of antioxidant and vasculoprotective nutrients (e.g., vitamin C and flavanones).^37^, ^38^ Moreover, the consumption of citrus fruit has been related to a lower risk of dementia.^39^ However, these benefits may be limited to fresh fruit or 100% fruit juices, since most commercial juices contain added sugars and have a similar energy density to soft drinks,^40^ which adversely impact health.^41^, ^42^ In the Framingham Heart Study, daily fruit juice intake was associated with lower brain volume and poorer episodic memory.^42^ In our study, the correlation analyses suggest that proline betaine mostly derived from juices.

Cyclo(prolyl‐valyl) and theobromine, derivatives of cocoa products, showed lower odds of CD, which is in accordance with previous observational results on chocolate intake and reduced CD,^43^ and with human intervention studies demonstrating that the consumption of cocoa flavanols can improve hippocampal vascular plasticity and reduce CD in healthy older persons.^44^, ^45^ We also found a putative marker of fish intake, CMPFP, to be associated with lower odds of CD. There is a large epidemiological literature linking fish consumption to lower CD^46^ and although CMPFP did not significantly correlate with fish intake in our sample, CMPFP was found increased after consuming fish‐rich Mediterranean and Nordic diets in two untargeted metabolomics studies,^47^, ^48^ and was recently confirmed as a potential biomarker of fish.^49^ Moreover, we found an unidentified ion highly correlated with (red) wine intake, to be associated with lower odds of CD. Accordingly, in a meta‐analysis, light‐to‐moderate alcohol intake, especially wine, was related to a lower risk of dementia.^50^

The signature included several endogenous metabolites previously reported to be dysregulated in metabolic syndrome or early in the course of AD, for example, the three acylcarnitines (myristoylcarnitine, undecanoylcarnitine/4,8 dimethylnonanoylcarnitine and lauroylcarnitine). Changes in plasma acylcarnitines are indicators of incomplete fatty acid beta‐oxidation in mitochondria and have been associated with overfeeding, high‐fat diets and metabolic syndrome.^51^, ^52^ In a rat model of cafeteria diet‐induced obesity, lauroylcarnitine was demonstrated to drive the polarization of macrophages towards the pro‐inflammatory “M1” phenotype, potentially mediating the pro‐inflammatory response to an unbalanced diet.^52^ Other studies reported variations of plasma medium‐chain acylcarnitines in AD and preclinical AD^53^, ^54^; however, the species involved has so far been inconsistent. A conjugated secondary bile acid (GDCA) and a glycerophospholipid (lysoPC(18:3)) were also associated with CD in our study. A disturbed cholesterol and lipid metabolism in the brain has long been suspected in AD.^55^ In particular, growing evidence suggests a link between increased blood levels of specific bile acids, including GDCA, and AD.^7^, ^56^ Considering the multiple roles of bile acids and their modulation by diet, exercise, and gut microbiota, their comprehensive profiling in future studies on CD would certainly be informative. Another compound of the signature, trimethyllysine (TML), may point toward a putative role of the gut‐brain axis in cognitive aging. TML is released from proteins degraded in lysosomes and is a precursor of carnitine. Interestingly, we observed a correlation (*r* = 0.44) between TML and the gut microbial metabolite of carnitine trimethylamine‐N‐oxide (TMAO), which has been linked to cardiovascular diseases.^57^ The effects of TML on health and aging are poorly documented, with the notable exception of a recent untargeted metabolomics study reporting an association of TML with increased risk of cardiovascular diseases.^58^

The findings regarding glucose and creatinine are in accordance with previous literature,^59^, ^60^ with a strong relation between diabetes and a greater risk of dementia. With respect to arginine, a few untargeted metabolomics studies reported an increased serum concentration in cognitively impaired older persons^8^ and a lower plasma arginine was one of the most significant metabolic differences in older adults with superior memory performance compared to subjects with normal performance or cognitive disorders.^61^ The inverse association between higher blood cortisol and slower CD was less expected as excessive levels of cortisol have been related with CD in older ages.^62^, ^63^, ^64^ However, studies have not all been consistent and for example, our findings are in accordance with a study reporting inverse associations between serum cortisol and cerebrospinal fluid biomarkers of AD.^65^

Our study has major strengths, including a population‐based prospective design with repeated cognitive assessment over up to 12 years, a rigorous case‐control sampling inspired by incidence density sampling (as recommended for nested case control studies), and the use of state‐of‐the art methods for both untargeted metabolomics and statistical modeling. However, some limitations should be stressed. New findings from this discovery study will require external validation. Only the accumulation of high‐quality metabolomics studies performed on independent prospective cohorts with complementary analytical platforms will allow developing a consolidated metabolic signature that can be used universally to predict later CD. Although we controlled for major possible confounders, we cannot rule out that other factors might have influenced the metabolic profiles, and residual confounding is still possible as in any epidemiological study. Moreover, data collected by dietary surveys is prone to measurement error, thus secondary analyses of correlations with food and nutrient intakes should be interpreted with caution.

In conclusion, we discovered in non‐demented participants from a prospective cohort, a serum signature of subsequent CD over 12 years, which increased the predictive ability beyond that of standard predictors by 13%. The top metabolites were derived from food/beverages and suggested a protective association of coffee, cocoa, and fish with CD, while possible negative effects of citrus juice and caffeine deserves further investigation in focused studies. The signature also revealed endogenous metabolites related to cardiometabolic health and known to be negatively affected by an unbalanced diet. Whether replicated in independent cohorts, our results will provide new targets for preventive/therapeutic nutritional strategies against CD in older persons.

## Conflict of Interest

The authors declare no conflict of interest.

## Supporting information

Supporting Information

Supporting Information

Supporting Information

Supporting Information

Supporting Information

Supporting Information

Supporting Information

Supporting Information

Supporting Information

Supporting Information
