# Supplementary material for: Diet‐Related Metabolites Associated with Cognitive Decline Revealed by Untargeted Metabolomics in a Prospective Cohort
Source: Mol Nutr Food Res. 2019 Jul 9;63(18):1900177. doi: 10.1002/mnfr.201900177 (PMC6790579; doi:10.1002/mnfr.201900177)
Supplement: Supplementary file 1 — Supporting Information [file MNFR-63-na-s010.docx]

**Supporting Information Figure S1. Flow chart of case-control study sample selection, the Three-City (3C) Bordeaux cohort**

**Exclusion criteria:**

- Prevalent dementia (n=43)
- No baseline serum sample available in the biobank (n=675)
- Not followed for cognition and dementia after baseline (n=93)

**Study population**

Participants with serum samples available in the biobank at baseline and with at least one repeated cognitive evaluation over 12 years

**n=1,336**

**Eligible sample for case-control sampling**

**n=1,293**

**3C Bordeaux sample at cohort inclusion**

**n=2,104**

**Initial case set**

The 220 participants with worst

slopes of cognitive decline

**n= 220**

**Initial control set***

Participants with cognitive decline below median value (i.e., slopes > median)

**n= 862**

**Case sample**

Cases of cognitive decline successfully matched to one control with same age at baseline, sex and level of education

**n= 209**

**Control sample**

Controls with cognitive decline below median value at dropout of their matched case

**n= 209**

***** For each case (with last cognitive measure in V), potential controls included participants: (i) followed at least up to V, (ii) not defined as a case in V, (iii) with a slower decline up to V (i.e., with a slope of cognitive change better than the median when only using repeated cognitive data up to V).
