# Supplementary material for: Diet‐Related Metabolites Associated with Cognitive Decline Revealed by Untargeted Metabolomics in a Prospective Cohort
Source: Mol Nutr Food Res. 2019 Jul 9;63(18):1900177. doi: 10.1002/mnfr.201900177 (PMC6790579; doi:10.1002/mnfr.201900177)
Supplement: Supplementary file 2 — Supporting Information [file MNFR-63-na-s001.docx]

**Supporting Information Figure S2: Spectral information supporting identification of ions of interest**

**Tentative identification of m/z 497.2383 as atractyligenin glucuronide**


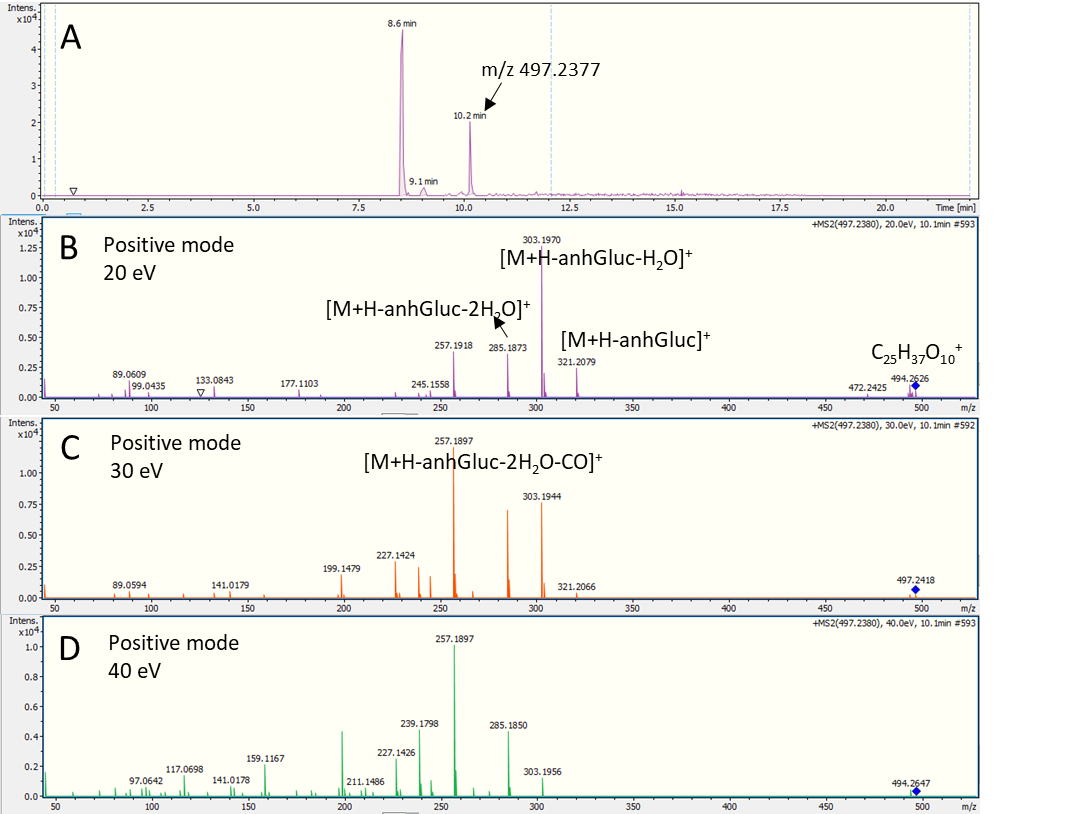


A) EIC of m/z 497.2377 at 10.2 min from UPLC-QTOF analysis of a serum sample of highest intensity. The MS/MS fragmentation of m/z 497.2377 at several collision energies of B) 20 eV, C) 30 eV and D) 40 eV, showed major fragments m/z 321.2079, 303.1970, 285.1873 and 257.1918, which became more intense as the induced collision energy increased from 20 to 40 eV. Fragments m/z 321.2079 and 303.1970 corresponds to the loss of an anhydrous glucuronide and further loss of water respectively. This fragmentation pattern matches with the fragments and in-source adducts described for atractyligenin glucuronide in ^[22]^. The small systemic difference in m/z and retention time is due to different LC-MS/MS analytical batches.

[22] J.A. Rothwell, Y. Fillâtre, J.-F. Martin, B. Lyan, E. Pujos-Guillot, L. Fezeu, S. Hercberg, B. Comte, P. Galan, M. Touvier, C. Manach, *PLoS One* **2014**, *9*.

**Tentative identification of m/z 269.1384 as 3-carboxy-4-methyl-5-pentyl-2-furanpropionic acid (CMPFP)**


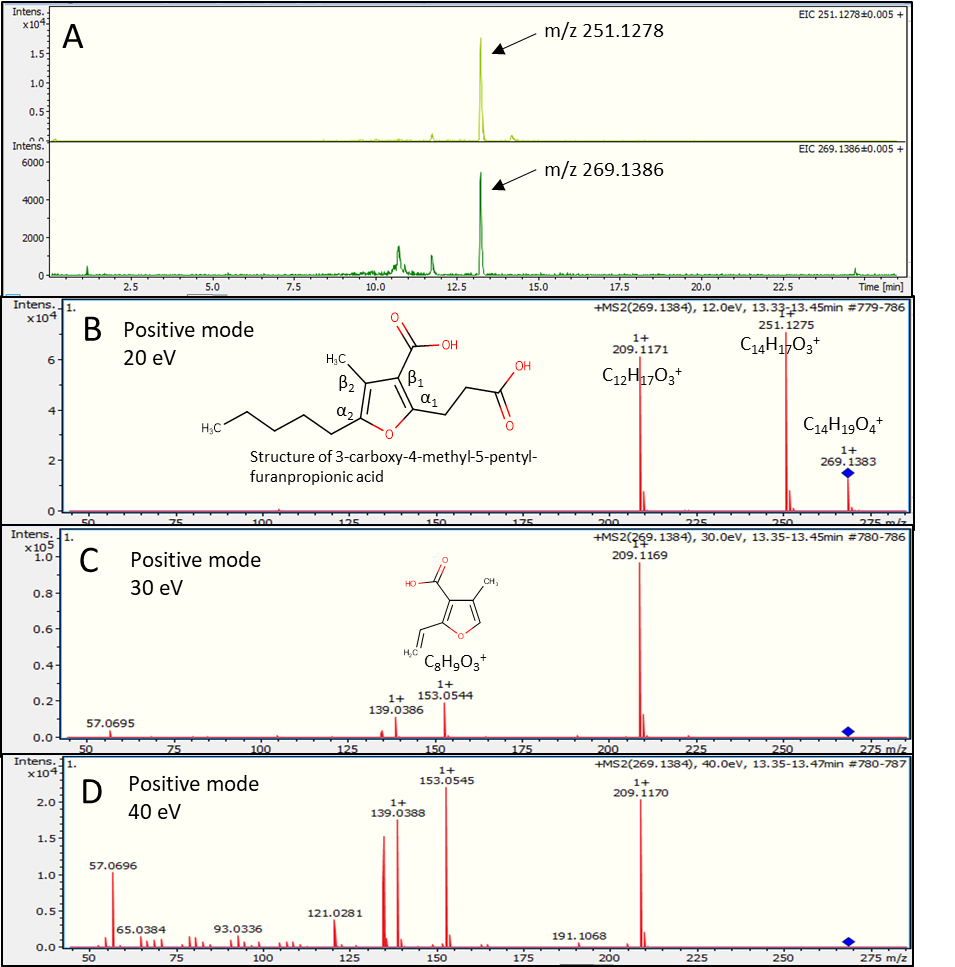


A) EIC of m/z 251.1278 and 269.1386 eluting at 13.37 min (m/z 269.1386 is annotated as the parent ion). The MS/MS fragmentation of m/z 269.1384 at several collision energies of B) 20 eV, C) 30 eV and D) 40 eV, showing major fragments m/z 251.1275, 209.1171, 153.0545, 139.0388 and 135.0439. The experimental m/z (269.1384) matches the reported mass (269.1383) in LipidMaps database and in previous studies^[23,24]^ supporting the tentative identification of m/z 269.1384 as CMPFP. The fragments match the predicted fragmentation pattern generated by Mass Frontier (Thermo Scientific), and are consistent with the experimental fragmentation of the analog CMPF. The small systemic difference in m/z and retention time is due to different LC-MS/MS analytical batches.

[23] J. Tovar, V.D. de Mello, A. Nilsson, M. Johansson, J. Paananen, M. Lehtonen, K. Hanhineva, I. Björck, *Molecular Nutrition & Food Research* **2017**, *61*, 1600552.

[24] I. Bondia‐Pons, J.A. Martinez, R. de la Iglesia, P. Lopez‐Legarrea, K. Poutanen, K. Hanhineva, M. de los Á. Zulet, *Molecular Nutrition & Food Research* **2015**, *59*, 711–728.

**Spectral data of m/z 129.0658 (unknown)**


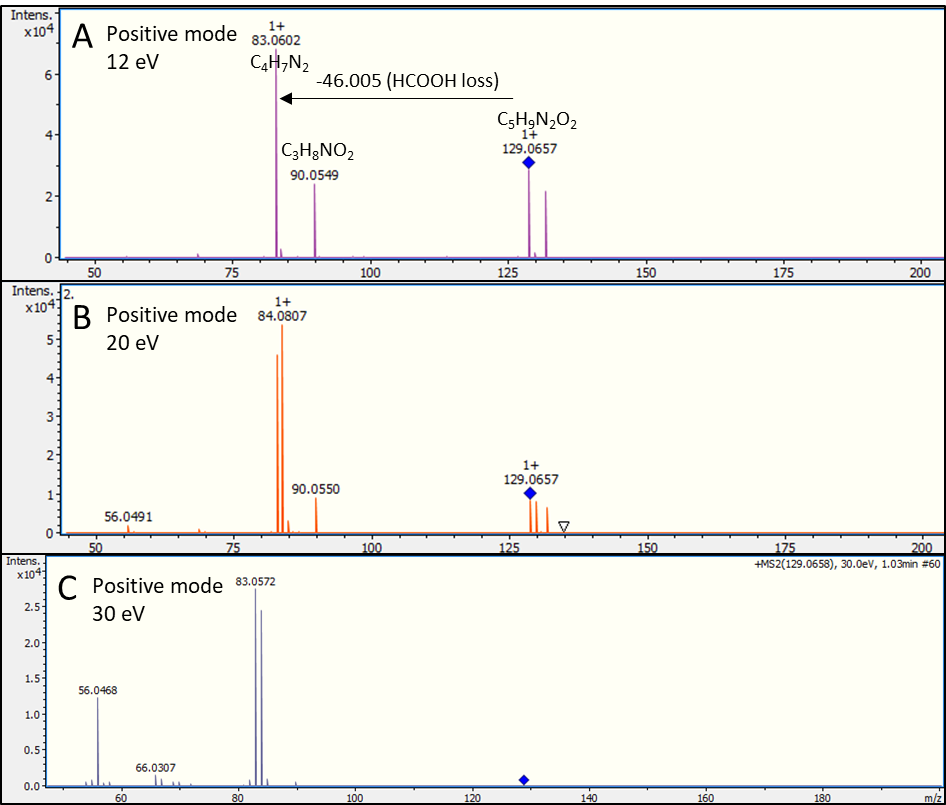


The MS/MS fragmentation of m/z 129.0657 at several collision energies of A) 12 eV, B) 20 eV and C) 30 eV, showed major fragments of m/z 90.0549, 84.080, 83.0602 and 56.0468.

**Spectral data of m/z 160.1331 (unknown)**


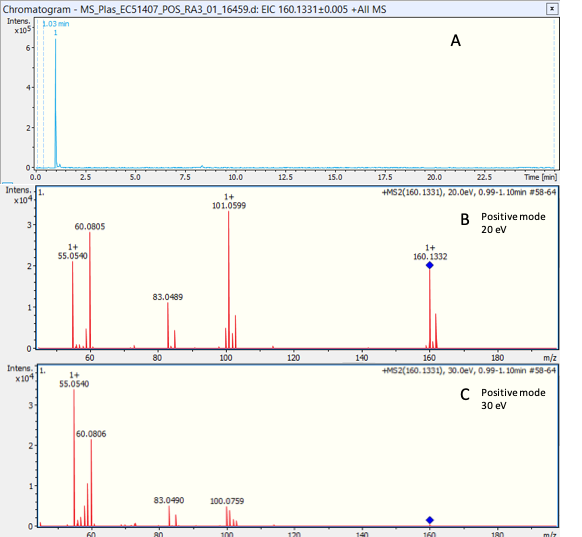


A) EIC of m/z 160.1331 eluting at 1.03 min. The MS/MS fragmentation pattern of m/z 160.1331 at several collision energies of A) 20 eV and B) 30 eV, showed major fragments of m/z 101.0599, 83.0489, 60.0805 and 55.0540 using a mass tolerance window of ±1.5 ppm.

**Spectral data of m/z 271.2056 (unknown)**


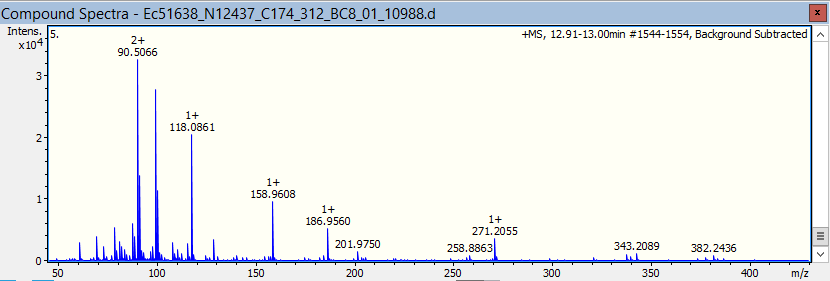


MS spectra of m/z 271.2056 (eluting at 12.99 min). MS/MS experiments were not carried out for this ion because of the lack of stability of this compound.

# Identification of m/z 197.1284 as cyclo(prolyl-valyl)

Fragmentation spectra of A) cyclo(prolyl-valyl) standard (eluting at 8.06 min) and B) m/z 197.1284 in sample (8.04 min) at collision energy 20 eV, and similarly at 30 eV (C-D), confirming m/z 197.1284 as cyclo(prolyl-valyl) as level l identification.

# Identification of m/z 383.1161 as glucose


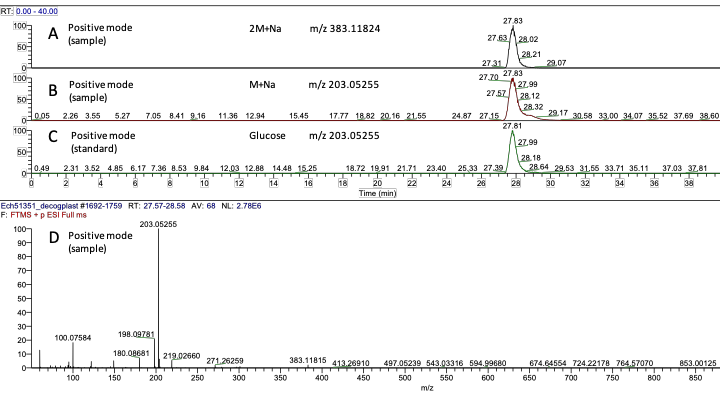


Through spectral annotation, m/z 203.0525 was identified as the sodium adduct [M+Na]^+^ of glucose [M+H]^+^ (m/z 181.0708), and m/z 383.1168 was identified as the 2M+Na adduct. Validation of m/z 383.1182 as glucose was confirmed with further analysis with on a chiral column with a commercial standard. Similar retention times were observed for A) m/z 383.1168 and B) 203.0525 from a sample and C) m/z 203.0525 (glucose standard) at 27.8 min.

**Spectral data of m/z 159.0276/137.0458 (unknown)**


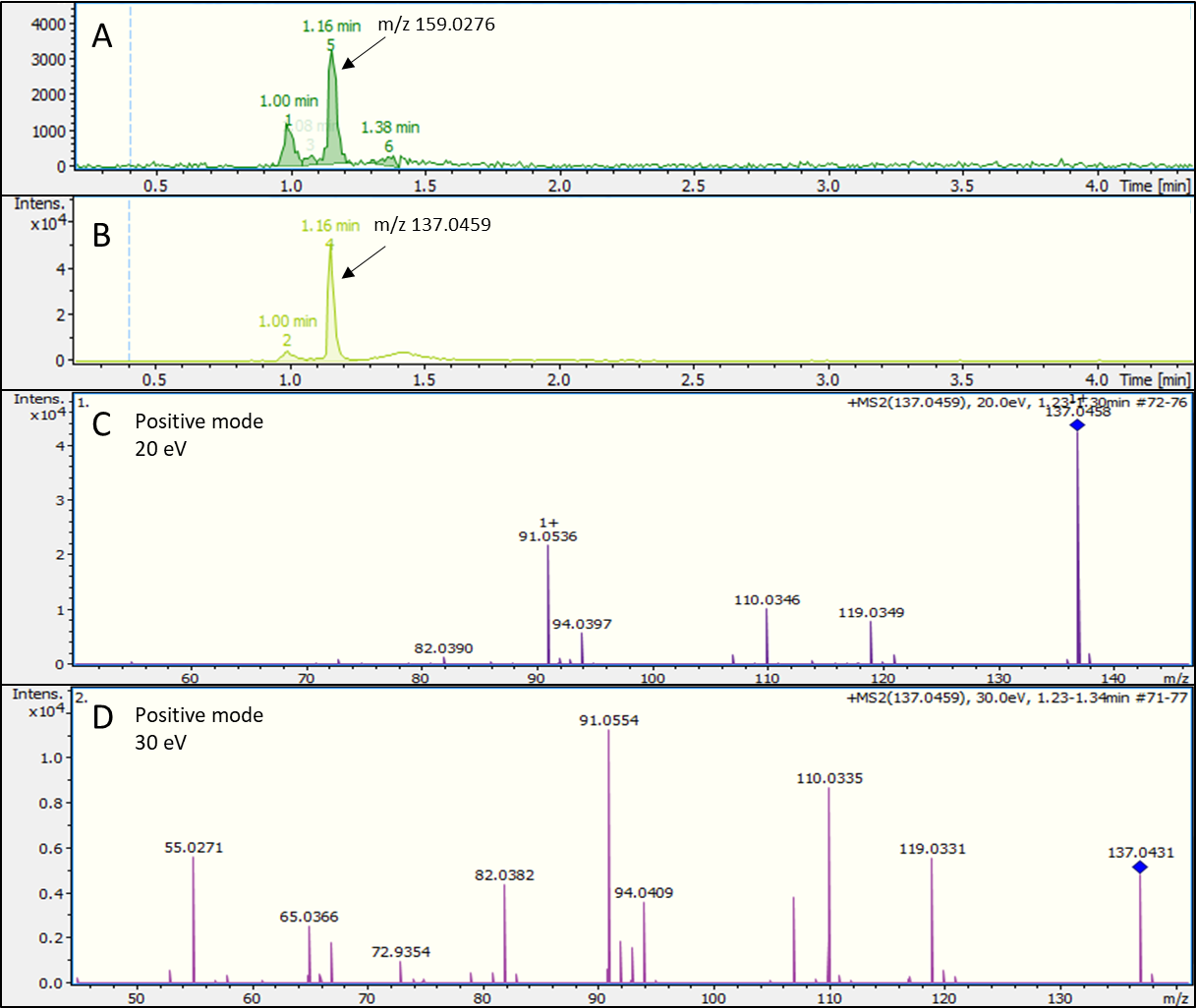


A) EIC of m/z 159.0275 and B) m/z 137.0459 eluting at 1.16 min. Through spectral annotation, m/z 159.0275 was identified as the sodium adduct [M+Na]^+^ of m/z 137.0459 [M+H]^+^. The MS/MS fragmentation of m/z 137.0459 at several collision energies of C) 20 eV and D) 30 eV showed major fragments of m/z 91.0536, 110.0346, 119.0331, 82.0382 and 55.0271, which increased in intensity as the induced collision energies increased. The fragmentation pattern was very similar to published spectra of hypoxanthine (C_5_N_4_N_4_O) in Metlin and m/zCloud except for the unexplained fragment ion m/z 91.0536. Analysis of the standard of hypoxanthine excluded this hypothesis because of different retention times.

# Identification of m/z 211.1441 as cyclo(leucyl-prolyl)

Fragmentation spectra of A) cyclo(leucyl-prolyl) standard (eluting at 9.2 min) and B) m/z 211.1450 in sample (9.2 min) at collision energy 20 eV, and similarly at 40 eV (C-D), confirming m/z 211.1441 as cyclo(leucyl-prolyl) as level l identification.

**Tentative identification of m/z 518.3242 as 1-linolenoyl-*sn*-glycero-3-phosphocholine (lysoPC(18:3))**


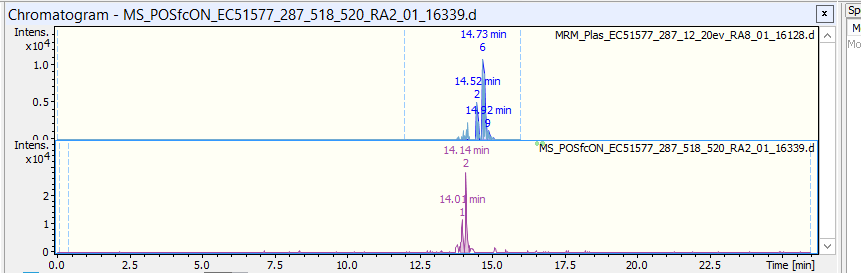

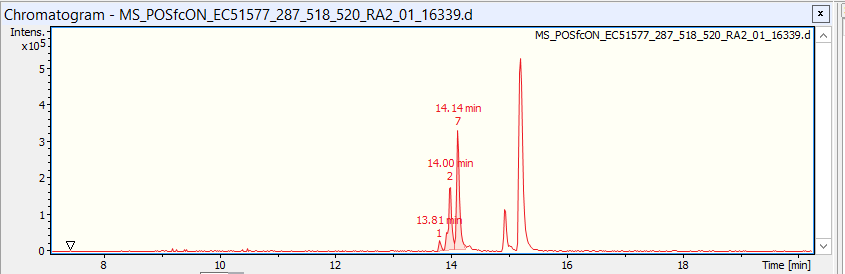

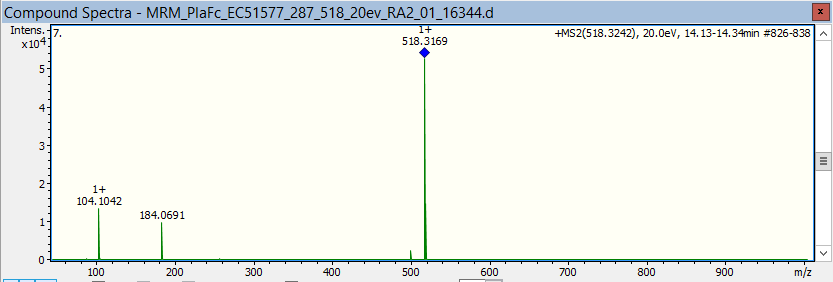

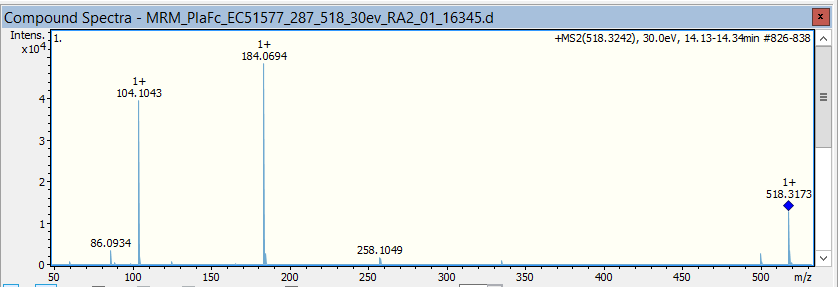


A

B

C

D

Positive mode

20 eV

Positive mode

30 eV

C_26_H_49_NO_7_P^+^

m/z 287.6257

m/z 518.3242

Phosphocholine fragment

C_5_H_15_NO_4_P^+^

Choline head group

C_5_H_12_N^+^

A) EIC of m/z 287.6257 and B) m/z 518.3242 eluting at 14.14 min. Through spectra annotation, m/z 518.3247 was observed as the parent ion of m/z 287.6256, a 2+ charged ion, sharing similar peak shapes and retention times. Fragmentation of m/z 518.3242 at C) 20 and D) 40 eV produced fragments 86.0934, 104.1042 and 184.0691, which are characteristic fragments of phosphatidycholines and previously reported in ^[25]^. The small systemic difference in m/z and retention time is due to different LC-MS/MS analytical batches.

[25] S. Suárez-García, L. Arola, A. Pascual-Serrano, A. Arola-Arnal, G. Aragonès, C. Bladé, M. Suárez, *J. Chromatogr. B Analyt. Technol. Biomed. Life Sci.* **2017**, *1055*–*1056*, 86–97.

**Identification of m/z 330.2639 as undecanoylcarnitine / 4,8 dimethylnonanoylcarnitine**

A

B

A) EIC of m/z 330.2639 eluting at 10.89 min analysed on the LTQ Orbitrap and B) MS/MS fragmentation at 40 eV produced the major fragments m/z 302.0632 and 85.0286.

**Spectral data of m/z 245.0768 (unknown)**


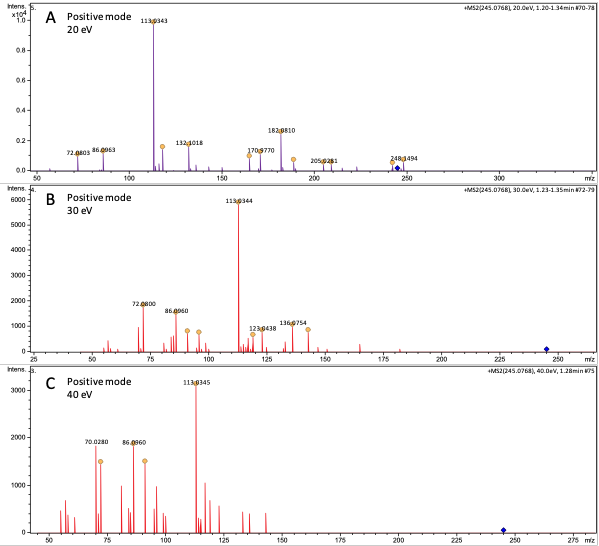


Fragmentation spectra of m/z 245.0768 (RT=1.15 min) at collision energies of A) 30 eV and B) 40 eV with a major fragment of m/z 113.0345 The ion shares similar monoisotopic mass to uridine and pseudouridine (C_9_H_12_N_2_O_6_), however upon confirmation with commercial standards, did not share similar fragmentation spectra (C, D) to pseudouridine (m/z 245.0767, 1.27 min) or uridine (m/z 245.0766 and 4.06 min).

**Spectral data of m/z 256.6796/512.3519 (unknown)**


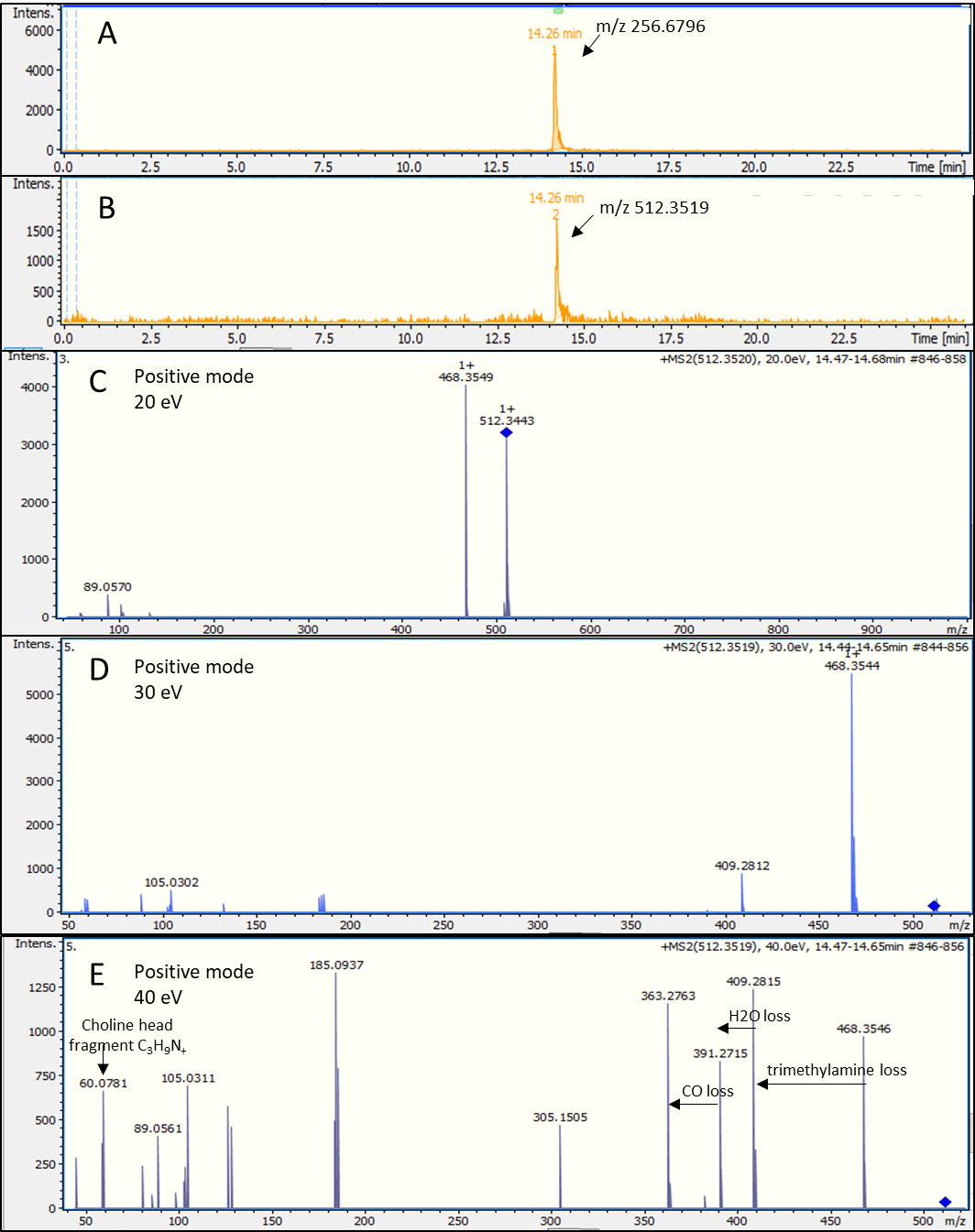


1. EIC of m/z 256.6796 and B) m/z 512.3519 eluting at 14.26 min; m/z 256.6796 was annotated as a 2+ charged ion with m/z 512.3519 (C_30_H_47_N_3_O_4_) being the parent ion, sharing similar peak shapes and retention times. The fragmentation spectra at collision energies of C) 20 eV, D) 30 eV and E) 40 eV produced the major ions m/z 512.3443, 468.3549, 409.2812, 363.2763 and 185.0937.

**Spectral data of m/z 96.0444 (unknown)**

**
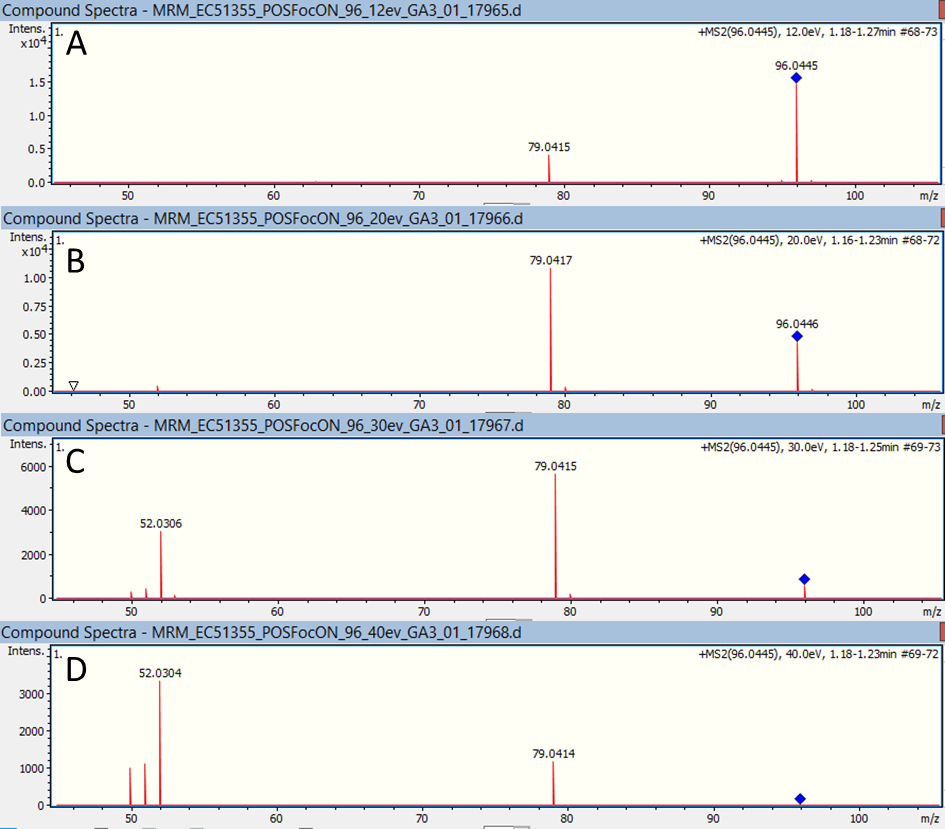
**

Fragmentation spectra of m/z 96.0444 at collision energies of A) 12 eV, B) 20 eV, C) 30 eV and D) 40 eV.
