# Supplementary material for: Diet‐Related Metabolites Associated with Cognitive Decline Revealed by Untargeted Metabolomics in a Prospective Cohort
Source: Mol Nutr Food Res. 2019 Jul 9;63(18):1900177. doi: 10.1002/mnfr.201900177 (PMC6790579; doi:10.1002/mnfr.201900177)
Supplement: Supplementary file 3 — Supporting Information [file MNFR-63-na-s002.docx]

**Supporting Information Figure S3: Heatmap of correlations between the 22 ions from the serum signature and food/nutrient intakes**

**
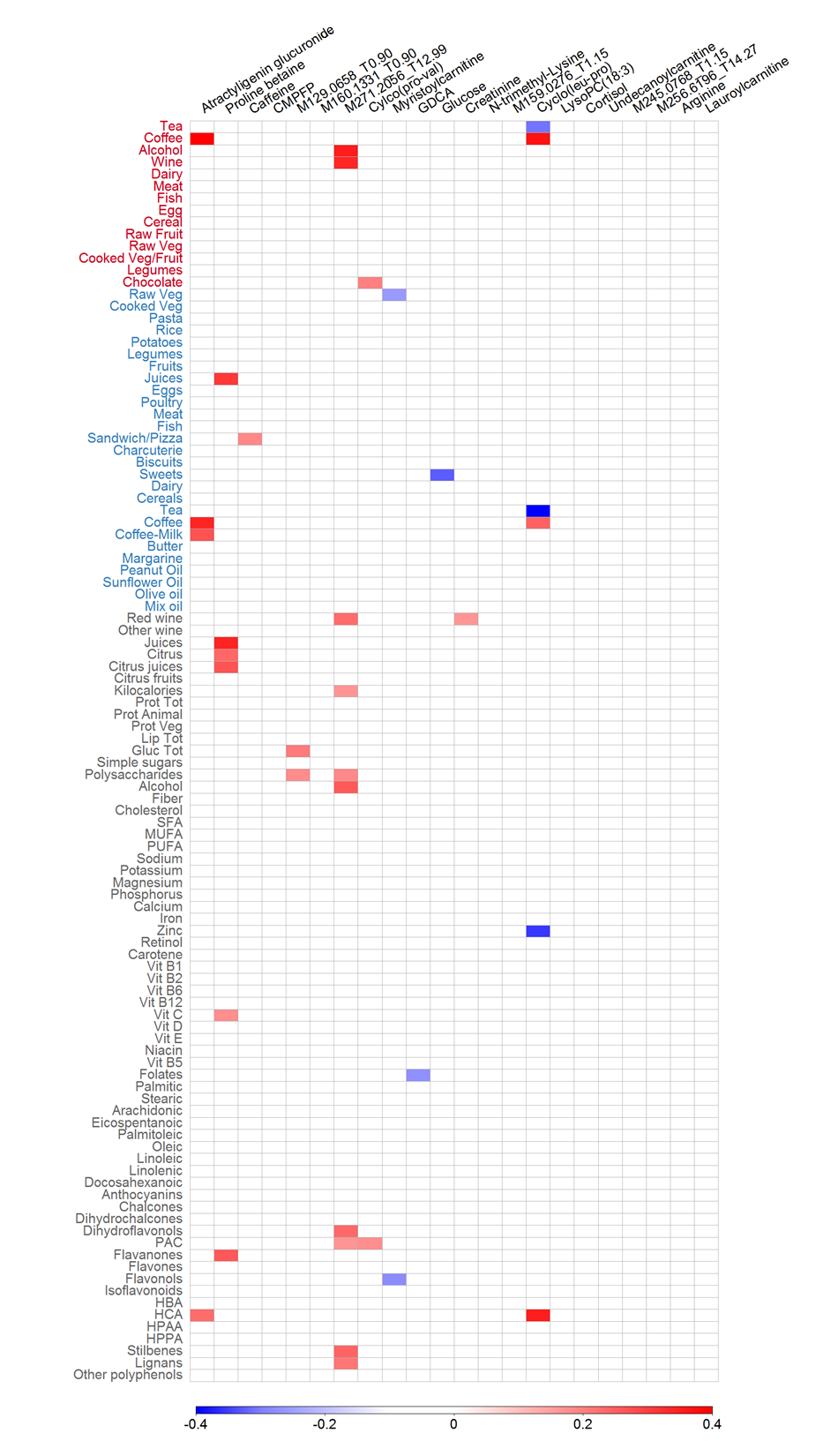
**

Foods/nutrients are colored according to the dietary assessment method used: brief Food Frequency Questionnaire (FFQ) ascertained at baseline in the entire 3C cohort including the n=418 participants from our case-control study (red), comprehensive FFQ (blue) and 24h dietary recall (grey) administered concomitantly in a subsample from 3C Bordeaux in 2001–2002, including n=351 participants from our case-control study.

*CMPFP, 3-Carboxy-4-methyl-5-pentyl-2-furanpropionic acid; GDCA, Glycodeoxycholic acid-3-glucuronide; LysoPC(18:3), 1-linolenoyl-sn-glycero-3-phosphocholine; SFA, Saturated fatty acid; MUFA, Monounsaturated fatty acid; PUFA, Polyunsaturated fatty acid; PAC, Proanthocyanidins; HBA, Hydroxybenzoic acid; HCA, Hydroxycinnamic acid; HPAA, Hydroxyphenylalaninic acid; HPPA, Hydroxyphenylpropanoic acid.*
