# Supplementary material for: Diet‐Related Metabolites Associated with Cognitive Decline Revealed by Untargeted Metabolomics in a Prospective Cohort
Source: Mol Nutr Food Res. 2019 Jul 9;63(18):1900177. doi: 10.1002/mnfr.201900177 (PMC6790579; doi:10.1002/mnfr.201900177)
Supplement: Supplementary file 4 — Supporting Information [file MNFR-63-na-s003.docx]

**Supporting Information Figure S4: Network map illustrating the relationship between coffee, hydroxycinnamates and serum metabolites**


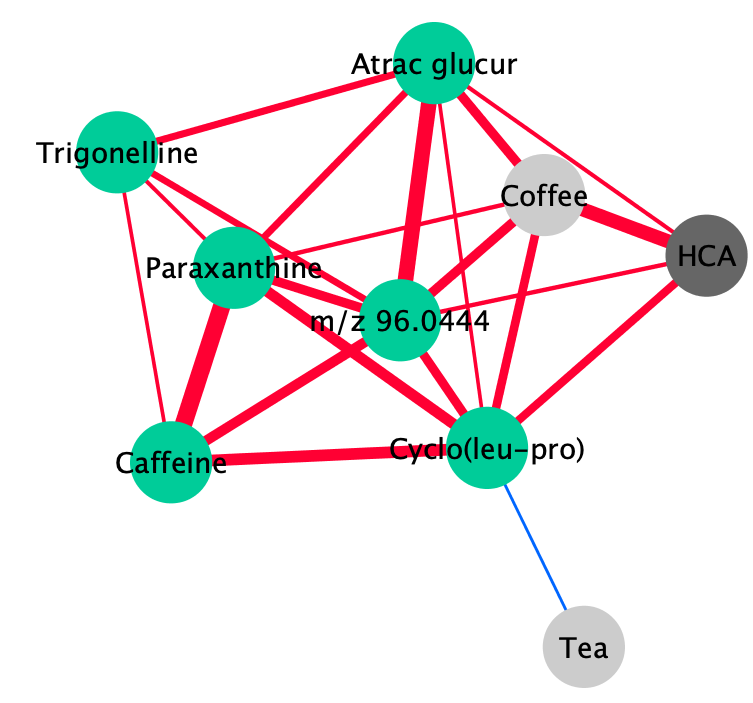


Width of the edges is proportional to the Pearson correlation coefficient amongst the relative abundance of the metabolites themselves or with relevant food groups (the thicker the edge, the stronger the correlation with the neighbouring metabolite or food group). Edge colours ― and ― denote positive and negative correlations respectively. Nodes are coloured ● for ions, ● for food groups, and ● for nutrient classes. Only correlations that were significant after correction for multiple testing (using the False Discovery Rate) and correlations whose absolute value was >0.20 are displayed. Intake of HCA was estimated using the Phenol Explorer database on polyphenol content in foods (phenol-explorer.eu).^[12,13]^

*Atrac glucur, atractyligenin glucuronide; Cyclo(leu-pro), Cyclo(leucyl-prolyl); HCA, Hydroxycinnamic acids.*

[12] V. Neveu, J. Perez-Jiménez, F. Vos, V. Crespy, L. du Chaffaut, L. Mennen, C. Knox, R. Eisner, J. Cruz, D. Wishart, A. Scalbert, *Database (Oxford)* **2010**.

[13] J.A. Rothwell, J. Perez-Jimenez, V. Neveu, A. Medina-Remón, N. M’Hiri, P. García-Lobato, C. Manach, C. Knox, R. Eisner, D.S. Wishart, A. Scalbert, *Database (Oxford)* **2013**.
