# Supplementary material for: Diet‐Related Metabolites Associated with Cognitive Decline Revealed by Untargeted Metabolomics in a Prospective Cohort
Source: Mol Nutr Food Res. 2019 Jul 9;63(18):1900177. doi: 10.1002/mnfr.201900177 (PMC6790579; doi:10.1002/mnfr.201900177)
Supplement: Supplementary file 5 — Supporting Information [file MNFR-63-na-s004.docx]

**Supporting information Table S1: Parameters used in metabolomic data pre-processing (Galaxy4Metabolomics workflow)**

|  | **Parameters** | **History** |
| --- | --- | --- |
| **xcms.xcms.Set** | method | centWave |
|  | ppm | 5 |
|  | peakwidth | 5, 20 |
|  | signal/noise threshold | 5 |
|  | m/z diff | 0.01 |
|  | peak limits method | smoothed 2nd derivative |
|  | prefilter | 3, 500 |
|  | noise filter | 0 |
| **xcms.group** | method | density |
|  | bandwidth | 10 |
|  | minfrac | 0.1 |
|  | mz width | 0.01 |
|  | max | 10 |
| **xcmc.retcor** | method | peakgroups |
|  | smooth | loess |
|  | extra | 1 |
|  | missing | 16 (15 blanks + 1 sample) |
|  | span | 0.8 |
|  | family | gaussian |
|  | plot type | mdeven |
| **xcms.group** | method | density |
|  | bandwidth | 8 |
|  | minfrac | 0.1 |
|  | mz width | 0.01 |
|  | max | 10 |
| **xcms.**  **fillpeaks** | method | chrom |
| **CAMERA.**  **annotate** | convert retention times (seconds) into minutes | YES |
|  | num_digits (for mass) | 8 |
|  | sigma | 6 |
|  | perfwhm | 1 |
|  | max charge | 3 |
|  | maxiso | 2 |
|  | minfrac | 0.3 |
|  | ppm | 5 |
|  | m/z | 0.015 |
|  | intval | Maxo * |
|  | max peaks | 100 |

|  | | **Parameters** | **History** |
| --- | --- | --- | --- |
| **CAMERA.**  **annotate**  (continued) | | quick mode | false |
|  |  | polarity | positive |
|  |  | cor_eic_th | 0.75 |
|  |  | pval | hcs |
|  |  | calcCis | yes |
|  |  | calcIso | yes |
|  |  | calcCaS | no |
|  |  | find Adducts | 2 |
|  |  | number of conditions | two or more |
|  |  | eicmax | 0 |
|  |  | eicwidth | 0 |
|  |  | value | 1.) Maxo |
|  |  | height | 480 |
|  |  | width | 640 |
|  |  | mzdec | 4 |
|  |  | sortpval | no |
| (performed manually) | Datasets of maxo and intb intensity values were merged into one sheet. Intensities of blanks and samples were re-calculated for obtaining new fold (mean of samples divided by mean of blanks) that was inserted into VMD file. | | |
| **Generic filter** | number of ions | | 8828 |
|  | retention time extremity: <0,04 & > 22 min | | 7110 |
|  | remove features where fold of sample/blank <2 | | 3416 |
| **Determine**  **batch**  **correction** | factor of interest | | batch |
|  | advanced options | | hide |
| **Batch correction** | regression model | | loess |
|  | span | | 1 |
|  | null values | | consider as null |
|  | factor of interest | | batch |
| **Quality metrics** | coefficient of variation | | yes |
|  | type of CV cal | | ratio btw pool & sample CV |
|  | threshold | | 1.25 |
|  | advanced parameters | | use default |
| **Generic  filter** | pool/sample CV: >1.25 | | 3136 |
| (performed manually) | number of ions | | 3136 |
|  | keep ions present **in ≥80%** of pools in 4 out of 4 batches | | 1219 |
|  | test presence: **30%** | | 1136 |

|  | **Parameters** | **History** |
| --- | --- | --- |
| **Metabolite correlation analysis**  **(MCA)** | function to be used | sorting your table and doing correlation analysis |
|  | correlation threshold for pcgroup | 0.8 |
|  | choice of the correlation method | pearson |
|  | cytoscape correlation threshold | 0.75 |
| **Redundancy filters**  (performed manually) | Remove redundant ions (from MCA) | 655 |
|  | remove isotopes with each new pcgrouping [M+2, M+3] |  |
|  | remove ions influenced by instrumental/batch analysis effect | 606 |
|  | Remove isotopes and their possible adducts/fragments, ions with <4000 maximum intensity and any remaining ions affected by batch correction | 301 |

*Zero value was considered for all peaks not initially detected.
