# Supplementary material for: Diet‐Related Metabolites Associated with Cognitive Decline Revealed by Untargeted Metabolomics in a Prospective Cohort
Source: Mol Nutr Food Res. 2019 Jul 9;63(18):1900177. doi: 10.1002/mnfr.201900177 (PMC6790579; doi:10.1002/mnfr.201900177)
Supplement: Supplementary file 6 — Supporting Information [file MNFR-63-na-s005.docx]

**Supporting information Table S2. Average intakes in detailed food groups and in nutrients of cases of cognitive decline and controls with slower cognitive decline among the participants from a case-control study matched for age, gender and education, nested within the 3C Bordeaux cohort who answered a comprehensive dietary survey in 2001-2002.***

|  | **Cases** | **Controls** |
| --- | --- | --- |
| **Food groups intake (number of servings/week)** |  |  |
| Raw vegetables and salad | 8.9 (4.7) | 9.3 (6.0) |
| Cooked vegetables | 10.5 (4.2) | 9.9 (4.1) |
| Pasta | 1.9 (1.5) | 1.9 (1.4) |
| Rice | 1.1 (1.1) | 1.1 (1.2) |
| Potatoes | 2.5 (1.5) | 2.5 (1.7) |
| Legumes | 0.5 (0.5) | 0.6 (0.7) |
| Fruit | 10.7 (5.4) | 10.7 (5.8) |
| Juices | 1.9 (3.8) | 1.5 (2.9) |
| Eggs | 1.5 (1.1) | 1.5 (1.1) |
| Poultry | 1.7 (1.2) | 1.8 (1.1) |
| Meat | 4.7 (2.4) | 4.6 (2.3) |
| Fish and seafood | 2.8 (1.7) | 2.7 (1.7) |
| Pizza, sandwich, salted pie | 0.4 (0.8) | 0.5 (1.0) |
| Charcuterie | 1.5 (2.0) | 1.6 (2.2) |
| Biscuits, cakes | 2.3 (4.1) | 2.0 (3.0) |
| Sweets | 8.6 (6.6) | 8.8 (6.2) |
| Dairy products | 15.7 (6.5) | 16.0 (7.1) |
| Cereals, bread | 18.6 (5.9) | 18.3 (4.9) |
| Tea | 2.6 (4.4) | 2.6 (4.6) |
| Coffee | 7.3 (5.1) | 7.8 (6.2) |
| **Energy intake (kcal/day)** | 1690.1 (542.2) | 1725.4 (545.3) |
| **Macronutrient intake (g/day)** |  |  |
| Proteins | 74.4 (25.7) | 74.6 (25.5) |
| Carbohydrates | 190.0 (69.8) | 194.5 (62.8) |
| Saturated fat | 26.2 (13.2) | 27.0 (15.0) |
| Monounsaturated fat | 21.0 (11.2) | 21.5 (11.2) |
| Polyunsaturated fat | 8.0 (5.2) | 8.7 (6.7) |
| **Micronutrient intake (g/day)** |  |  |
| Omega-3 polyunsaturated fatty-acids | 1.3 (1.4) | 1.2 (1.3) |
| Vitamin D | 1.9 (3.1) | 1.4 (1.8) |
| Folate | 263.7 (136.1) | 296.8 (150.5) |
| Vitamin C | 76.9 (58.0) | 93.4 (68.8) |
| Vitamin E | 6.2 (3.9) | 6.5 (4.0) |
| β-carotene | 3964.6 (7069.7) | 4160.9 (5965.6) |
| Total polyphenols | 952.8 (563.9) | 1004.8 (567.0) |

Values are mean (SD).

^*^among 418 participants of the initial case-control study, n=351 answered the comprehensive FFQ (n=164/209 cases and n=187/209 controls) and n=359 answered the 24h recall (n=167/209 cases and n=192/209 controls).
