# Supplementary material for: Diet‐Related Metabolites Associated with Cognitive Decline Revealed by Untargeted Metabolomics in a Prospective Cohort
Source: Mol Nutr Food Res. 2019 Jul 9;63(18):1900177. doi: 10.1002/mnfr.201900177 (PMC6790579; doi:10.1002/mnfr.201900177)
Supplement: Supplementary file 7 — Supporting Information [file MNFR-63-na-s006.docx]

**Supporting InformationTable S3: Detailed description of the 22 serum metabolites associated with subsequent cognitive decline in a case-control study nested within the 3C Bordeaux cohort.**

| **Compound** | **Molecular formula** | **RT (min)** | **Measured mass [M+H]+**  **(Da)** | **Mass error (mDa)** | **Fragments and/or adducts^1^**  **(Da)** | **Identification level^2^** | **Collision energy (eV)^3^** | **Main fragments found in MS/MS^3^**  **(Da)** |
| --- | --- | --- | --- | --- | --- | --- | --- | --- |
| Atractyligenin glucuronide | C25H36O10 | 10.11 | 497.2383 | 0.24 | 519.2194 [M+Na]+,  514.2650 [M+NH4]+,  **497.2383 [M+H]+,**  479.2270 [M+H-H2O]+, 321.2062 [M+H-anhydrogluc]+, 303.1950 [M+H-gluc]+ | 2 | 20-40 | 497.2380, 321.2079, 303.1970, 285.1873, 257.1918, 89.0594 |
| Proline betaine | C7H13NO2 | 0.96 | 144.1018 | -0.04 | 166.0847 [M+Na]+,  **144.1018 [M+H]+** | 1 | 20-40 | 144.1017, 98.0962, 84.0814 72.0808, 58.0650 |
| Caffeine | C8H10N4O2 | 8.09 | 195.0876 | 0.03 | 217.0696 [M+Na]+,  **195.0876 [M+H]+** | 1 | 22 | 195.0873, 138.0659, 110.0709, 83.0604, 69.0449 |
| 3-Carboxy-4-methyl-5-pentyl-2-furanpropionic acid (CMPFP) | C14H20O5 | 13.25 | 269.1382 | -0.02 | 291.1198 [M+Na]+,  269.1382 [M+H]+,  **251.1278 [M+H-H2O]+,**  209.1173 [M+H-C2H2O]+ | 2 | 12-40 | 269.1384, 209.1170, 153.0545, 139.0388, 135.0439, 121.0281, 57.0696 |
| Unknown | C5H8N2O2 | 0.90 | 129.0658 | -0.04 | **129.0658 [M+H]+,**  83.0601 {M+H-CH2O2]+ | 4 | 12-40 | 129.0657,  84.0807, 83.0572, 66.0307, 56.0468 |

| **Compound** | **Molecular formula** | **RT (min)** | **Measured mass**  **[M+H]+**  **(Da)** | **Mass error (mDa)** | **Fragments and/or adducts^1^**  **(Da)** | **Identification level^2^** | **Collision energy (eV)^3^** | **Main fragments found in MS/MS^3^**  **(Da)** |
| --- | --- | --- | --- | --- | --- | --- | --- | --- |
| Unknown | C8H17NO2 | 0.97 | 160.1331 | -0.01 | **160.1331 [M+H]+** | 4 | 12-40 | 160.1332, 101.0599, 100.0759, 83.0489, 60.0805,  55.0540 |
| Unknown | C19H26O | 12.99 | 271.2056 | -0.03 | **271.2056 [M+H]+** | 4 | 12-40 |  |
| Cyclo(prolyl-valyl) | C10H16N2O2 | 7.95 | 197.1284 | 0.02 | **197.1284 [M+H]+** | 1 | 12-40 | 197.1284, 169.1331, 154.0737, 141.1384, 124.1118, 98.0601, 100.0725, 72.0778, 70.0649 |
| Myristoylcarnitine (=Tetradecanoyl-carnitine) | C21H41NO4 | 13.12 | 372.3108 | 0.04 | **372.3108 [M+H]+** | 1 | 25* | 372.3108, 313.2369, 85.0285 |
| Glycodeoxycholic acid-3-glucuronide | C32H51NO11 | 11.69 | 626.3536 | 0.17 | 648.3349 [M+Na]+,  643.3797 [M+NH4]+,  **626.3536 [M+H]+** | 1 | 12-40  35* | 626.3536, 432.3103, 414.2996, 339.2679, 76.0391 |
| Glucose | C6H12O6 | 0.87 | 203.0525 | 0.14 | **383.1161 [2M+H]+,**  203.0526 [M+Na]+,  181.0708 [M+H]+ | 1 |  |  |
| Creatinine | C4H7N3O | 0.88 | 114.0660 | -0.10 | 227.1253 [2M+H]+,  136.0482 [M+Na]+,  **114.0660 [M+H]+** | 1 | 18.6 | 114.0663, 86.0713, 72.0443 |

| **Compound** | **Molecular formula** | **RT (min)** | **Measured mass**  **[M+H]+**  **(Da)** | **Mass error (mDa)** | **Fragments and/or adducts^1^**  **(Da)** | **Identification level^2^** | **Collision energy (eV)^3^** | **Main fragments found in MS/MS^3^**  **(Da)** |
| --- | --- | --- | --- | --- | --- | --- | --- | --- |
| N-Trimethyl-L-lysine | C9H20N2O2 | 0.79 | 189.1597 | 0.01 | 211.1416 [M+Na]+,  **189.1597 [M+H]+** | 1 | 12-40  40* | 189.1345, 172.1080, 144.1131, 130.0862, 84.0807 |
| Unknown | C5H4N4O | 1.15 | 137.0458 | 0.07 | **159.0276 [M+Na]+,**  137.0458 [M+H]+ | 4 | 12-40 | 137.0458, 119.0349, 110.0346, 94.0397, 91.0536, 82.0390,  72.9354,  65.0366,  55.0271 |
| Cyclo(leucyl-prolyl) | C11H18N2O2 | 8.78 | 211.1441 | 0.02 | **211.1441 [M+J]+** | 1 | 20-40 | 211.1397, 114.0881, 98.0597, 84.9597, 70.0657 |
| 1-linolenoyl-*sn*-glycero-3-phosphocholine (lysoPC(18:3)) | C26H48NO7P | 14.09 | 518.3242 | 0.16 | 518.3242 [M+H]+,  287.6256 **[M+2H]2+** | 2 | 12-80 | 184.0694, 104.1043, 86.0934 |
| Cortisol | C21H30O5 | 10.80 | 363.2166 | 0.03 | **363.2166 [M+H]+** | 1 | 40* | 363.2268, 327.1947, 267.1272, 241.1586, 163.1116 |

| **Compound** | **Molecular formula** | **RT (min)** | **Measured mass**  **[M+H]+**  **(Da)** | **Mass error (mDa)** | **Fragments and/or adducts^1^**  **(Da)** | **Identification level^2^** | **Collision energy (eV)^3^** | **Main fragments found in MS/MS^3^**  **(Da)** |
| --- | --- | --- | --- | --- | --- | --- | --- | --- |
| Undecanoylcarnitine/4,8 dimethylnonanoylcarnitine | C18H35NO4 | 11.58 | 330.2639 | 0.10 | **330.2639 [M+H]+** | 3 | 40* | 330.2638, 320.0751  302.0632, 85.0286 |
| Unknown | C9H12N2O6 | 1.15 | 245.0768 | 0.06 | 267.0590 [M+Na]+,  **245.0768 [M+H]+** | 4 | 12-40 | 245.0768  182.0810,  132.1019,  118.0863,  113.0343,  86.0963,  72.0803 |
| Unknown | C30H45N3O4 | 14.27 | 512.3519 | 3.62 | 512.3519 [M+H]+,  **256.6796 [M+2H]2+** | 4 | 12-40 | 512.3433, 468.3549, 409.2812, 391.2715, 363.2763, 305.1505, 185.0937, 105.0311,  89.0561,  60.0781 |
| L-Arginine | C6H14N4O2 | 0.81 | 175.1189 | 0.01 | 197.1008 [M+Na]+,  **175.1189 [M+H]+** | 1 | 40* | 175.1191, 158.0926, 130.0978, 116.071, 70.0654 |
| Lauroylcarnitine (=Dodecanoylcarnitine) | C19H37NO4 | 12.23 | 344.2795 | 0.06 | **344.2795 [M+H]+** | 1 | 40* | 344.2793, 285.2063, 85.0286, 60.0809 |

Metabolites ordered by decreasing frequency of selection across bootstraps. **^1^**Masses in bold refer to ions selected in the conditional logistic regression models. **^2^**Levels of identification are as follows: level 1: compounds identified by matching of masses, MS/MS fragmentation and retention time to commercially available standards; level 2: compounds tentatively identified by matching masses and MS/MS fragmentation to spectra in databases and literature; level 3: compounds identified by masses and spectra similarities to a compound class; level 4: unknown compounds. **^3^**MS/MS analysis was performed with LC-QTOF using multiple collision energies. *MS/MS fragmentation performed on Orbitrap using higher-energy collisional dissociation (HCD). Da, Daltons; RT, Retention time.
