# Supplementary material for: Diet‐Related Metabolites Associated with Cognitive Decline Revealed by Untargeted Metabolomics in a Prospective Cohort
Source: Mol Nutr Food Res. 2019 Jul 9;63(18):1900177. doi: 10.1002/mnfr.201900177 (PMC6790579; doi:10.1002/mnfr.201900177)
Supplement: Supplementary file 8 — Supporting Information [file MNFR-63-na-s007.docx]

**Supporting Information Method S1**

**Study population**

To build a case-control study of cognitive decline, we utilized individual slopes of cognitive change estimated by a linear mixed model. Our primary outcome was the change in a composite score of global cognition assessing various cognitive domains. The composite score was defined at each follow-up as the average of Z-scores of five neuropsychological tests: (i) the Mini-Mental State Examination (which assesses global cognitive performance),^[1]^ (ii) the Benton Visual Retention Test (assessing visual working memory and attention),^[2]^ (iii) the Isaac’s Set Test (assessing verbal fluency),^[3]^ (iv) the Trail-Making Test part A (which reflects processing speed),^[4]^ and (v) the Trail-Making Test part B (which reflects executive functioning).^[4]^ The repeated composite cognitive scores were normalized using a latent process mixed model (to ensure Gaussian assumption)^[5]^ before being entered as a dependent variable in the linear mixed model. The model included an intercept (that represented the level of composite cognitive score at baseline), a slope (that represented the annual change in scores over time) and both a random intercept and a random slope to account for inter-individual variability (as well as a binary indicator for the first cognitive assessment).

In order to obtain individual slope estimates as close as possible to the data, we ran a separate model for each dropout group (19%, 14%, 15% and 51% of participants with a last visit at V= 4, 7, 10 or 12 years of follow-up respectively). From these models, we extracted individual slopes (as the sum of estimated fixed effect + predicted individual random effect) and identified the 220 participants with the worst slopes of cognitive decline up to the dropout time (with a maximum of 12 years). Furthermore, for each case (with last cognitive measure in V), we randomly selected a control among participants: (i) followed at least up to V, (ii) not defined as a case in V, (iii) with a slower decline up to V (i.e., with a slope of cognitive change better than the median when only using repeated cognitive data up to V); (iv) of same age (±3 years), sex and educational level (<vs ≥ secondary school). Overall, 209 participants with greater cognitive decline were successfully individually matched to one control with slower cognitive decline, leading to a total sample size of n=418 subjects.

**Dietary data**

Dietary habits were primarily assessed using a brief FFQ ascertained at baseline (1999-2000) in the entire 3C cohort.^[6]^ For specific foods/nutrients not ascertained with this FFQ (e.g., polyphenols), we used a comprehensive FFQ and a 24h dietary recall administered in a subsample from 3C Bordeaux in 2001–2002. Among the 1,811 3C Bordeaux participants examined at the first follow-up visit in 2001-2002, 1,796 individuals completed the dietary assessment, including 351 participants from our case-control study. The brief qualitative FFQ administered at baseline assessed habitual intakes of 14 food/beverages categories (dairy products, meat, fish, egg, cereals, raw fruits, raw vegetables, cooked fruits/vegetables, legumes, chocolate, tea, coffee, alcohol and wine). Frequency of consumption was recorded in six classes: never, <1 time/week, 1 time/week, 2 to 3 times/week, 4 to 6 times/week, and ≥ 7 times/week. Dietary intakes obtained with this brief questionnaire showed reasonable correlations with intakes estimated with the comprehensive FFQ administered in 2001-2002 (e.g., ρ=0.52 for fish intake), and we previously found significant associations between dietary habits assessed with this questionnaire and cognitive decline and the risk of dementia.^[7,8]^

The comprehensive FFQ administered in 2001-2002 assessed the consumption of 40 categories of foods and non-alcoholic beverages for the three main meals and three between-meals snacks.^[9]^ Frequencies of consumption were recorded in 11 classes and transformed into discrete variables as follows: 0 for never or less than once a week, 0.25 for once a month, 0.5 for twice a month, 0.75 for three times a month, 1 for once a week, and from 2 for twice a week to 7 for seven times a week. This coding was used to estimate the number of usual weekly servings of each of the 148 food items, ranging from 0 to 42 (with a maximum of six meals a day). The number of glasses of alcohol per week was also recorded. The food items were then aggregated into 20 food and beverage groups: fresh fruits and compotes; all kinds of meat, excluding poultry; fish and seafood; pizzas, sandwiches, quiches, and salted pies; biscuits, cakes, cookies, and Viennese pastries; all kinds of sweet products; all kinds of dairy products; cereals and bread. All other food groups included a single item: raw vegetables and salad; cooked vegetables; pasta; rice; potatoes; pulses; eggs; charcuterie; alcohol; coffee; tea. As with the brief FFQ, dietary habits assessed from the comprehensive FFQ in Bordeaux were associated with cognitive decline and the risk of dementia in the cohort.^[7,8]^ Intakes of nutrients and polyphenols (including hydroxycinnamates, flavanones and proanthocyanidins, stilbenes) were ascertained through a 24-hour dietary recall administered concomitantly to the comprehensive FFQ in 2001-2002. The 24h recall consisted in reporting all meals and beverages consumed during the 24h preceding the interview (excluding week-end meals).^[10]^ The quantity of each food item was assessed using a manual of portion size photographs.^[11]^ Individual daily intakes in macro and micronutrients, including main polyphenols classes, were estimated by multiplying food/beverage intakes by their nutrient content using standard nutrient composition databases (eg, Phenol-Explorer^[12,13]^ for polyphenols), as detailed in previous publications.^[10,14]^ The 24h dietary recall was also used to estimate the habitual consumption of specific food groups (eg, citrus intake, red wine intake) when the information was not available in any of the two FFQs.

[1] M.F. Folstein, S.E. Folstein, P.R. McHugh, *Journal of Psychiatric Research* **1975**, *12*, 189–198.

[2] A.L. Benton, Manuel du test de rétention visuelle: applications cliniques et expérimentales, Editions du Centre de psychologie appliquée, Paris **1953**.

[3] B. Isaacs, A.T. Kennie, *The British Journal of Psychiatry* **1973**, *123*, 467–470.

[4] R.M. Reitan, *Percept Mot Skills* **1958**, *8*, 271–276.

[5] C. Proust-Lima, V. Philipps, J.-F. Dartigues, D.A. Bennett, M.M. Glymour, H. Jacqmin-Gadda, C. Samieri, *Statistical Methods in Medical Research* **2017**, 096228021773965.

[6] S. Larrieu, L. Letenneur, C. Berr, J.F. Dartigues, K. Ritchie, A. Alperovitch, B. Tavernier, P. Barberger-Gateau, *J Nutr Health Aging* **2004**, *8*, 497–502.

[7] P. Barberger-Gateau, C. Raffaitin, L. Letenneur, C. Berr, C. Tzourio, J.F. Dartigues, A. Alpérovitch, *Neurology* **2007**, *69*, 1921–1930.

[8] C. Samieri, M.-C. Morris, D.A. Bennett, C. Berr, P. Amouyel, J.-F. Dartigues, C. Tzourio, D.I. Chasman, F. Grodstein, *American Journal of Epidemiology* **2018**, *187*, 933–940.

[9] C. Samieri, M.-A. Jutand, C. Féart, L. Capuron, L. Letenneur, P. Barberger-Gateau, *Journal of the Academy of Nutrition and Dietetics* **2008**, *108*, 1461–1471.

[10] C. Féart, M.A. Jutand, S. Larrieu, L. Letenneur, C. Delcourt, N. Combe, P. Barberger-Gateau, *Br. J. Nutr.* **2007**, *98*, 1046–1057.

[11] S. Hercberg, M. Deheeger, P. Preziosi, Collectif, Portions alimentaires : Manuel photos pour l’estimation des quantités, Economica, Paris **2002**.

[12] V. Neveu, J. Perez-Jiménez, F. Vos, V. Crespy, L. du Chaffaut, L. Mennen, C. Knox, R. Eisner, J. Cruz, D. Wishart, A. Scalbert, *Database (Oxford)* **2010**.

[13] J.A. Rothwell, J. Perez-Jimenez, V. Neveu, A. Medina-Remón, N. M’Hiri, P. García-Lobato, C. Manach, C. Knox, R. Eisner, D.S. Wishart, A. Scalbert, *Database (Oxford)* **2013**.

[14] S. Lefevre-Arbogast, D. Gaudout, J. Bensalem, L. Letenneur, J.-F. Dartigues, B.P. Hejblum, C. Feart, C. Delcourt, C. Samieri, *Neurology* **2018**, *90*, 1979–1988.
