# Supplementary material for: Diet‐Related Metabolites Associated with Cognitive Decline Revealed by Untargeted Metabolomics in a Prospective Cohort
Source: Mol Nutr Food Res. 2019 Jul 9;63(18):1900177. doi: 10.1002/mnfr.201900177 (PMC6790579; doi:10.1002/mnfr.201900177)
Supplement: Supplementary file 9 — Supporting Information [file MNFR-63-na-s008.docx]

**Supporting Information Method S2: Sample preparation and untargeted LCMS metabolomics.**

***Sample preparation***

Serum aliquots collected from the participants at the baseline of the study were protein-precipitated with acidified methanol (0.1% formic acid, 250 µL) and centrifuged for 10 min at 13,000 rpm, 4°C. The supernatant (250 µL) was collected, added with 250 µL acetonitrile and stored at -80°C prior to LCMS analysis.

***LCMS analysis***

An U3000 UHPLC system (Thermo Scientific, France) was coupled to a high-resolution Bruker Impact HD ll UHR-QTOF (Bruker Daltonics, Germany) fitted with an electrospray ionisation (ESI) source and a lock-mass sprayer to ensure accuracy. UHPLC separation was performed on a Waters HSS T3 column (150 x 2.1 mm, 1.8 µm) at a flow rate of 0.4 mL/min at 30°C and with an injection volume of 5 µL. Sample injection order was randomised into four batches based on age, BMI and sex, and matched case-control pairs were arranged in the same batch to avoid inter-batch variation. Quality controls obtained by mixing 10 μL of all extracted samples were injected at the beginning and end of each batch and every 10 samples to monitor stability of the analytical system and allow signal drift and batch effect correction. Mobile phases A and B were MilliQ water (18.2MΩ) and acetonitrile with 0.1% formic acid respectively. The gradient elution was 0% B (2 min), 0-100% B (13 min), 100% B (7 min), 100-0% B (0.1 min) and 0% B (3.9 min for re-equilibration). The mass resolution of the mass spectrometer was 50,000 and mass accuracy ranged from 0.8-2 ppm. Samples were analysed in the positive ionisation mode. Capillary and end plate offset voltages were set at 2500 V and 500 V. The drying gas temperature was 200°C and nebulisation gas flow was 10 L/min. Mass spectrum data was acquired in full-scan mode over mass range 50-1000 m/z.

***Data processing workflow***

LCMS data was processed using the Galaxy online interface (WorkFlow4Metabolomics.org).^[15]^ Peak detection, grouping and retention time correction were performed using the XCMS module^[16]^ followed by annotation using the CAMERA module.^[17]^ Full details of the workflow history and additional processing steps are listed in **Supporting Information Table S1**. After quality checks and signal drift correction, ions that were not present in 80% of quality control pools per batch were discarded. In this study, we are interested in food-derived metabolites so features present in ≥30% of either case or control population were kept, to accommodate for inter-individual variability in dietary habits (the threshold is usually 80% in studies for endogenous markers). This resulted in a data matrix of 1136 ions being characterized by retention time, m/z and relative intensity. This matrix was used for all correlation analyses. The detected ions can correspond to a parent compound, or to adducts, isotopes or fragments of the same metabolite. Redundancy should be avoided for statistical analyses such as least absolute shrinkage and selection operator (LASSO) regression. Hence, highly correlated ions (>80% Pearson correlation) from the same metabolite within the same retention time cluster were removed using the Metabolite Correlation Analysis Galaxy module. A further filtering step removed ions with too high remaining variability and ions below a minimum intensity threshold (<4000), resulting in a final data matrix of 301 ions.

***Metabolite identification***

Initial annotation of the full scan mass spectra (Bruker DataAnalysis v4.3) was performed to identify the parent molecular ion and its fragments and adducts. Peak shapes, retention times and an in-house program built to recognize a custom list of typical fragments, adducts and neutral spectral losses were used. MS/MS analyses were performed on the parent ions in selected serum samples using the Bruker Impact II UHR-QTOF at collision energies of 12, 20, 30 and 40 eV with a mass unit tolerance width of ±1.5 or ±3 mDa and same conditions as for the full scan experiments. MS/MS analyses were also performed in an Accela chromatograph coupled to a LTQ Orbitrap Velos MS equipped with an ESI source (Thermo Scientific, UK). Mass spectra were acquired in profile mode with a setting of 30 000 resolution at m/z 400. MS conditions were set as follows: capillary temperature, 375 ºC; sheath gas, 20 (arbitrary units); auxiliary gas, 10 (arbitrary units); sweep gas, 2 (arbitrary units); source voltage, 3.5 kV. Furthermore, MS/MS spectra were acquired at a resolving power of 15 000, using the higher-energy collisional dissociation (HCD) mode. Data analysis was achieved using XCalibur software v2.0.7 (Thermo Fisher Scientific).

Spectral data were compared to an internal spectral library of >2500 standards and searched for matches in public databases including Human Metabolome Database (HMDB) (<http://www.hmdb.ca)>, PhytoHub (<http://phytohub.eu)>, METLIN (<https://metlin.scripps.edu)>, Dictionary of Food Compounds (<http://dfc.chemnetbase.com)>, LipidMaps (<http://www.lipidmaps.org)>, MzedDB (<http://maltese.dbs.aber.ac.uk:8888/hrmet/index.html)>, m/z Cloud ([www.mzcloud.org](http://www.mzcloud.org)) and ChEBI (<https://www.ebi.ac.uk/chebi)> with a mass tolerance window of 5 ppm. Custom-curated databases of known biomarkers of intake for specific foods, endogenous compounds associated with cognition, and the Bordeaux 3C medication list (top 50 frequently consumed medications amongst the 418 participants) were built to search hypotheses of identification with high biological plausibility. Mass Frontier (Thermo Scientific) was used to predict fragmentation pathways for hypotheses when no experimental MS/MS data were available.

Final identification was achieved after a combination of MS/MS analyses and checking for analytical and biological plausibility using public spectral databases and literature. When available, standards were purchased and analysed in the same conditions as samples. Cyclo(prolyl-valyl) was purchased from Santa Cruz Biotechnology, Inc (California, USA). Cyclo(leucyl-prolyl) and glycodeoxycholic acid-3-glucuronide were purchased from Toronto Research Chemicals (Ontario, Canada). Proline betaine was purchased from Extrasynthese (Genay, France). Caffeine, creatinine, N-trimethyl-L-lysine, glucose, L-arginine, cortisol, lauroylcarnitine and myristoylcarnitine were purchased from Sigma-Aldrich (Saint Quentin Fallavier, France).

Four levels of identification were reported:^[18]^ level 1 refers to compounds identified by matching of masses, MS/MS fragmentation pattern and retention time with commercially available standards; level 2 refers to compounds tentatively identified by matching of masses and MS/MS fragmentation pattern with spectra published in online libraries and/or in the literature; level 3 refers to compounds identified only by masses or spectra similarities to a compound class and literature knowledge; and level 4 refers to unknown compounds.

[15] F. Giacomoni, G. Le Corguillé, M. Monsoor, M. Landi, P. Pericard, M. Pétéra, C. Duperier, M. Tremblay-Franco, J.-F. Martin, D. Jacob, S. Goulitquer, E.A. Thévenot, C. Caron, *Bioinformatics* **2015**, *31*, 1493–1495.

[16] C.A. Smith, E.J. Want, G. O’Maille, R. Abagyan, G. Siuzdak, *Anal. Chem.* **2006**, *78*, 779–787.

[17] C. Kuhl, R. Tautenhahn, C. Böttcher, T.R. Larson, S. Neumann, *Anal Chem* **2012**, *84*, 283–289.

[18] L.W. Sumner, A. Amberg, D. Barrett, M.H. Beale, R. Beger, C.A. Daykin, T.W.-M. Fan, O. Fiehn, R. Goodacre, J.L. Griffin, T. Hankemeier, N. Hardy, J. Harnly, R. Higashi, J. Kopka, A.N. Lane, J.C. Lindon, P. Marriott, A.W. Nicholls, M.D. Reily, J.J. Thaden, M.R. Viant, *Metabolomics* **2007**, *3*, 211–221.
