# Supplementary material for: Diet‐Related Metabolites Associated with Cognitive Decline Revealed by Untargeted Metabolomics in a Prospective Cohort
Source: Mol Nutr Food Res. 2019 Jul 9;63(18):1900177. doi: 10.1002/mnfr.201900177 (PMC6790579; doi:10.1002/mnfr.201900177)
Supplement: Supplementary file 10 — Supporting Information [file MNFR-63-na-s009.docx]

**Supporting Information Method S3: Statistical analyses**

We used least absolute shrinkage and selection operator (LASSO) regression for matched studies to identify a set of metabolites associated with the odds of subsequent cognitive decline over up to 12 years.^[19]^ LASSO allows the selection of a subset of variables associated with an outcome in high-dimensional setting. LASSO relies on a penalization parameter that controls for the strength of selection by shrinking the coefficients toward zero (setting some to exactly zero thus performing variable selection); the optimal penalty (and the corresponding subset selection) was chosen by leave-pair-out cross-validation. Because of the case-control study design, we applied LASSO to a conditional logistic regression, allowing the selection of a subset of metabolites associated with the odds of being a case versus a control while taking into account the matching procedure (i.e., analysis conditioned on age at baseline, sex and level of education). The model was adjusted for BMI and the total number of medications regularly consumed (un-penalized variables). As LASSO regression may lead to unstable solutions, bootstrap resampling (i.e., random sampling with replacement from the initial sample) has been proposed to provide a more robust selection of variable.^[20]^ We therefore used a bootstrapped-version of LASSO-penalized conditional logistic regression, consisting in repeating the initial procedure on 1,000 bootstrapped samples. Finally, we intersected the resulting 1,000 selections of metabolites and ordered the metabolites by decreasing percentage of selection across bootstraps. Because a metabolite selected by LASSO in many bootstraps may be reliably associated with cognitive decline, we primarily focused on the metabolites selected in >40% of bootstraps (top-selected metabolites). The LASSO shrinkage causes the estimates of coefficients to be biased towards zero; one approach correcting the coefficients for their bias is to compute a standard un-penalized model including the set of selected metabolites.^[21]^ However, the variance of these estimates remains biased (because it neglects the uncertainty associated with the complex selection procedure for obtaining the subset of metabolites) and confidence intervals thus cannot be computed.

[19] R. Tibshirani, *Journal of the Royal Statistical Society, Series B* **1994**, *58*, 267–288.

[20] F.R. Bach, Bolasso: model consistent Lasso estimation through the bootstrap, in: Proceedings of the 25th International Conference on Machine Learning, **2008**, pp. 33–40.

[21] A. Belloni, V. Chernozhukov, *Bernoulli* **2013**, *19*, 521–547.
